# Parkinson Disease ‐Targeted Nanocapsules for Synergistic Treatment: Combining Dopamine Replacement and Neuroinflammation Mitigation

**DOI:** 10.1002/advs.202404717

**Published:** 2024-10-21

**Authors:** Ziyao Liu, Shijun Xiang, Bei Chen, Jian Li, Dingcheng Zhu, Hongjuan Xu, Shuo Hu

**Affiliations:** ^1^ Department of Nuclear Medicine Xiangya Hospital Central South University Changsha 410008 China; ^2^ Key Laboratory of Biological Nanotechnology of National Health Commission Xiangya Hospital, Central South University Changsha 410008 China; ^3^ College of Material Chemistry and Chemical Engineering Key Laboratory of Organosilicon Chemistry and Material Technology Ministry of Education Hangzhou Normal University Hangzhou 311121 China; ^4^ National Clinical Research Center for Geriatric Diseases Xiangya Hospital Central South University Changsha 410008 China

**Keywords:** carrier‐free delivery system, macromolecule delivery, neurotransmitter delivery, Parkinson disease, synergistic therapy

## Abstract

Parkinson's disease (PD) is characterized by dopamine (DA) neuron loss and neuroinflammation. This study develops carrier‐free nanocapsules (NCs) for targeted delivery of DA and catalase (CAT) to the PD brain, addressing both DA depletion and neuroinflammation simultaneously. The NCs are engineered by DA and 4‐formylphenylboronic acid co‐loading with cRGD‐modified CAT (CAT‐cRGD) and surface‐modifying with Angiopep‐2 (Ang). Ang targets the blood‐brain barrier (BBB), enhancing brain delivery, while cRGD targets upregulated integrin receptors in the PD‐affected BBB. The NCs showed a 1.4‐fold increase in parkinsonian brain targeting efficiency compared to normal mice. In PD mice models, NCs demonstrated a stable increase in learning and memory, enhanced locomotor activity, and improved motor coordination. DA supplementation significantly enhanced dopaminergic signaling, increasing DA levels 1.8‐ and 3.5‐fold in the striatum and substantia nigra, respectively. Additionally, delivered CAT effectively reduced neuroinflammation by mitigating endoplasmic reticulum stress, slowing disease progression, and protecting DA from oxidation. This innovative approach using PD‐targeted NCs represents a synergistic strategy for PD treatment, combining symptomatic relief with disease progression intervention.

## Graphical abstract

This study presents Parkinson's Disease (PD)‐targeted carrier‐free nanocapsules (NCs) for the simultaneous delivery of dopamine and catalase to the PD brain, addressing both dopamine depletion and neuroinflammation. By integrating symptomatic and etiological treatments, these NCs offer a comprehensive and innovative approach to PD therapy, providing a potential breakthrough in disease management.

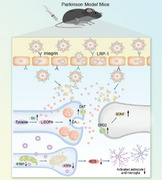

## 1 Introduction

Parkinson disease (PD) is the second most common neurodegenerative disorder, characterized by motor symptoms such as rigidity, bradykinesia, and non‐motor symptoms including cognitive decline and mood disorders. The global burden of PD is rapidly increasing, with an estimated 13 million cases expected by 2040.^[^ ^1^ ^]^ The pathology of PD is primarily caused by the irreversible loss of dopaminergic neurons in the substantia nigra and the accumulation of misfolded α‐synuclein (α‐syn).^[^ ^2^ ^]^ The prion‐like behavior of misfolded α‐syn allows them to spread from one neuron to others and across brain regions. The aggregation of misfolded α‐syn in the endoplasmic reticulum (ER) and mitochondria leads to increased ER stress, mitochondrial dysfunction, and oxidative stress.^[^ ^3^ ^]^ These alterations trigger neuroinflammation, which is a key factor in the pathogenesis of PD. The loss of dopaminergic neurons and aggregation of misfolded α‐syn induce neuroinflammation, which in turn can further oxidize dopamine (DA) and promote α‐syn misfolding, creating a self‐perpetuating cycle.^[^ ^4^ ^]^

Current treatments for PD primarily focus on alleviating symptoms by either directly or indirectly increasing brain dopamine levels.^[^ ^5^ ^]^ While dopaminergic medications provide symptomatic relief and improve quality of life, they are unable to halt or slow down disease progression. Additionally, the blood‐brain barrier (BBB) presents a significant hurdle for drug delivery, as it effectively blocks 98% of small molecules and nearly all macromolecules from entering the brain parenchyma.^[^ ^6^ ^]^ This insufficient delivery of therapeutic agents requires higher doses or more frequent administration over time. Therefore, efficient delivery of therapies to the brain is a critical aspect of PD treatment. Nanotechnology offers promising solutions to overcome the BBB and improve therapeutic delivery with minimal side effects. Surface‐modified nanoparticles can specifically penetrate the BBB through various mechanisms, such as adsorptive‐mediated, carrier‐mediated, and receptor‐mediated transcytosis.^[^ ^7^ ^]^ Several nanodrugs have been developed for the treatment of PD. However, these nanodrugs still have limitations, as most of them primarily focus on reducing neuroinflammation.^[^ ^8^, ^9^, ^10^, ^11^ ^]^ The single‐target strategies, failing to address the complex, network‐based nature of PD pathogenesis. Additionally, the use of external nanocarriers, such as inorganic nanoparticles, raises safety concerns due to potential accumulation in neuron microenvironment and interference with microglial clearance.^[^ ^12^ ^]^ These limitations highlight the urgent need for developing biocompatible carrier‐free nanodrugs that offer a synergistic, multi‐target approach to PD therapy.

**Scheme 1:**
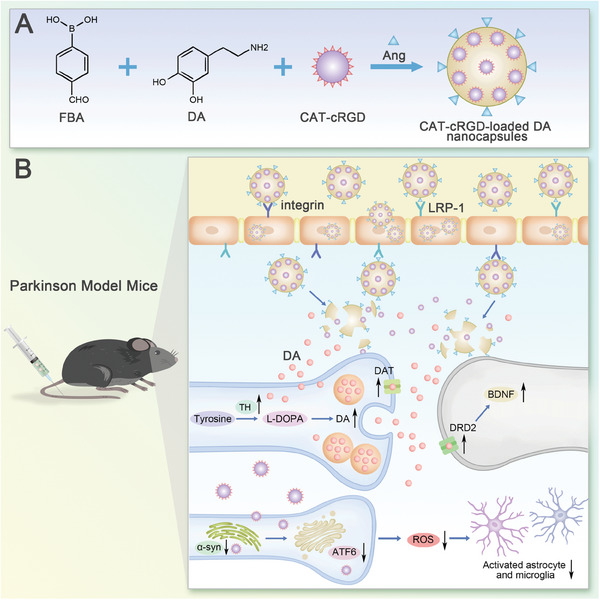
Schematic Illustration of PD‐Brain Targeting Nanocapsules for Synergistic Therapy. A) The carrier‐free NCs were fabricated FBA and DA, followed by the loading of CAT‐cRGD and surface modification with Ang. B) Upon intravenous (i.v.) administration, the dual‐targeting NCs traverse the BBB by exploiting the interaction between Ang and theLRP‐1 expressed on the BBB. Moreover, cRGD specifically targets to the upregulated integrin receptors on the BBB under PD conditions, further enhancing the BBB penetration of the NCs. Once in the brain, the NCs augment DA signaling and elevate DA levels in the striatum and substantia nigra. Concurrently, the NCs attenuate the expression of α‐synuclein (α‐syn) and activating transcription factor 6 (ATF6), resulting in a reduction of reactive oxygen species (ROS) and neuroinflammation. TH: tyrosine hydroxylase, DAT: dopamine transporter, DRD2: dopamine D2 receptor, BDNF: brain‐derived neurotrophic factor.

Herein, we aimed to develop a biocompatible carrier‐free nanodrug for synergistic therapy for PD treatment. As shown in **Scheme** **1**, our innovative parkinsonian brain‐targeting nanocapsules (NCs) efficiently deliver DA to the brain parenchyma while simultaneously reducing neuroinflammation, offering a synergistic approach to PD therapy. The carrier‐free NCs are synthesized from DA and 4‐formylphenylboronic acid (FBA), ensuring biocompatibility and minimizing safety concerns associated with external carrier. The co‐loading catalase (CAT) modified with cyclo(CRGDfK) (cRGD) aims to decrease neuroinflammation. Furthermore, the modified cRGD binds to the increased expression of integrin receptors in the vascular endothelial cells of parkinsonian brains, creating PD‐targeted NCs.^[^ ^13^, ^14^, ^15^ ^]^ To improve brain targeting, Angiopep‐2 (Ang), which has a high affinity for low‐density lipoprotein (LDL)‐related protein‐1(LRP‐1) in the BBB, is adsorbed onto the surface of NCs, facilitating their penetration into the brain.^[^ ^16^, ^17^ ^]^ Our therapeutic NCs provide a dual‐target approach: 1) NCs efficiently transport DA into the brain, significantly alleviating disease symptoms; 2) encapsulated CAT intervenes in the progression of PD by reducing neuroinflammation. Additionally, CAT protects DA from oxidation, enhancing its therapeutic efficacy during delivery and within the lesion site. In summary, our study introduces a carrier‐free nanoformulation that addresses the limitations of current PD treatments. By exploiting the specific characteristics of the parkinsonian brain and incorporating a multi‐target approach, our NCs have the potential to revolutionize PD therapy, providing a more effective and comprehensive treatment option.

## 2 Results and Discussion

### 2.1 Preparation and Characterizations of Carrier‐Free Nanocapsules

The BBB presents a significant challenge in delivering therapeutic agents to the brain. However, the progression of PD leads to disease‐specific alterations in the BBB, such as increased angiogenesis and integrin expression, which can be exploited for targeted drug delivery. To enhance the delivery efficiency of our NCs, we modified CAT with cRGD (CAT‐cRGD), which specifically targets integrins overexpressed in the BBB during PD. Although there are no definitive studies identifying the specific type of integrin overexpressed in the BBB during PD, we utilized cRGD to broadly target various integrins.^[^ ^18^, ^19^, ^20^, ^21^ ^]^ This conjugation of cRGD to CAT was achieved using a previously described method,^[^ ^22^ ^]^ and the modification was confirmed by sodium dodecyl sulfate‐polyacrylamide gel electrophoresis, which showed an increase in molecular weight for the CAT‐cRGD compared to unmodified CAT (Figure S1, Supporting Information). The enzyme activity of CAT‐cRGD was demonstrated through the oxidation of 3,3′,5,5′‐tetramethylbenzidine (TMB) in the presence of hydrogen peroxide, indicating that the enzyme's activity was preserved post‐modification (Figure S1, Supporting Information).

**Figure 1:**
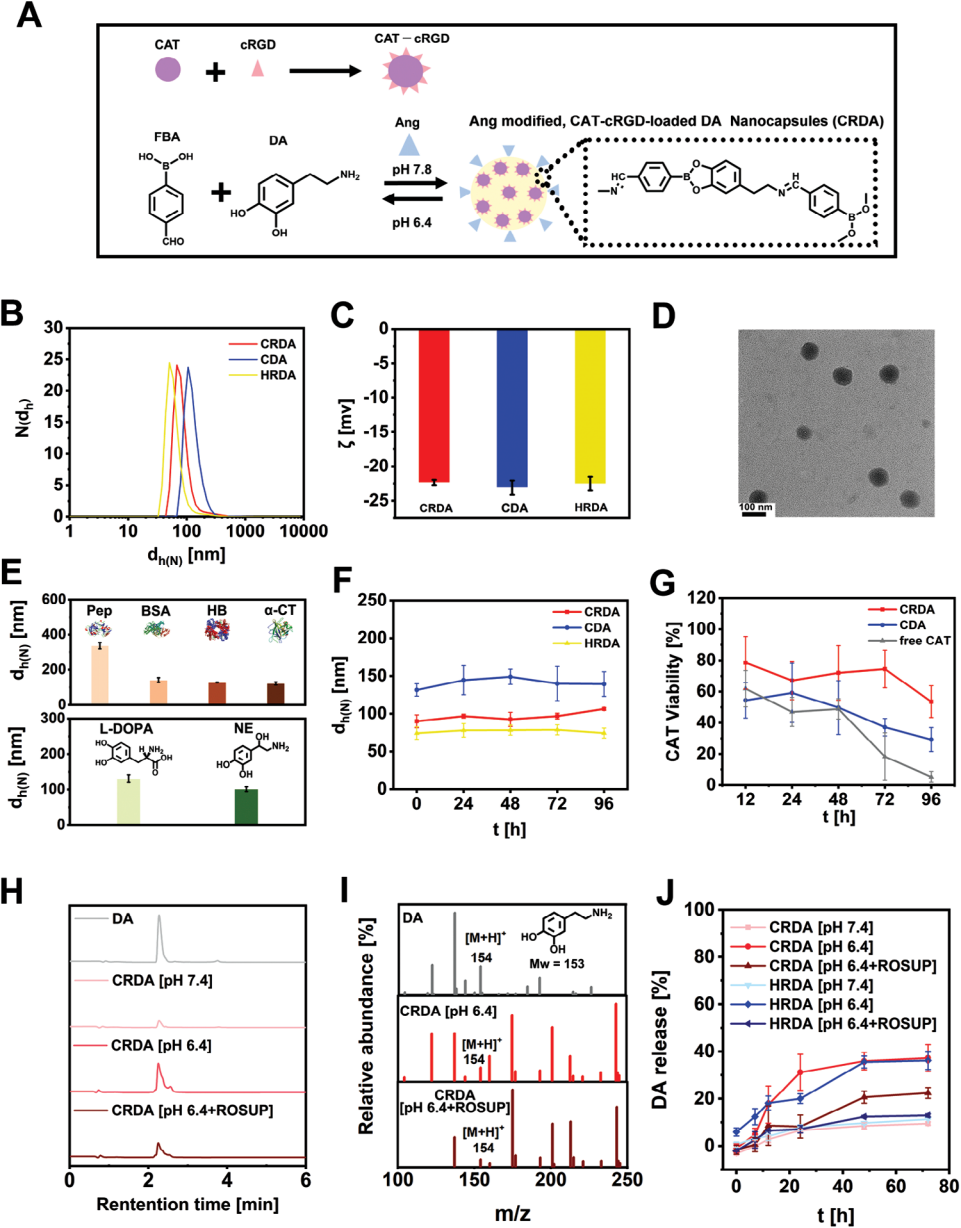
Construction and Characterization of Therapeutic Nanocapsules (NCs). A) Illustration of the synthetic process for creating Ang modified, CRDA. B) Size distribution N(d_h_) of CRDA, CDA, and HRDA at a concentration of C_NCs_ = 1 mg mL^−1^. C) Zeta potential measurements ζ for CRDA, CDA, and HRDA at the same concentration. D) Transmission electron microscopy (TEM) image showcasing the morphology of CRDA. E) Hydrodynamic sizes d_h(N)_ of NCs loaded with various proteins and built using different molecules (C_NCs_ = 1 mg mL^−1^). F) Stability analysis of the hydrodynamic size d_h(N)_ for three NCs in PBS over 96 h. G) CAT viability of CRDA, CDA, and free CAT at a concentration of C_CAT_ = 50 µg/mL over 96 h at room temperature, with data normalized to 100% viability at t = 0 h (*n* = 3). H) HPLC profiles of samples separated using an ultracentrifuge filter after 72 h incubation with CRDA under various conditions at a concentration of C_DA_ = 30 µg mL^−1^, alongside DA monomers at a concentration of 7.81 µg mL^−1^. I) Eluents collected at retention times between 2 and 2.5 min were further analyzed using electrospray ionisation mass spectrometry (ESI‐MS). J) Release profiles of DA from CRDA and HRDA under different conditions at a concentration of C_DA_ = 50 µg mL^−1^ (*n* = 3).

The carrier‐free‐ NCs was fabricated through the reaction FBA with DA at pH 7.8, simultaneously incorporating CAT‐cRGD (**Figure** **1**A). To further enhance brain delivery efficiency, Ang was adsorbed on the surface of NCs, known for its affinity to the LRP‐1 on the BBB. The changes in zeta potential verified the successful modification of Ang on the surface of NCs (Figure S2, Supporting Information). We designed three groups of NCs to comprehensively evaluate the impact of cRGD modification and CAT payload: Ang‐modified, CAT‐cRGD‐loaded DA NCs (CRDA), Ang modified, CAT‐loaded DA NCs (CDA), and Ang‐modified, HSA‐ cRGD ‐loaded DA NCs (HRDA). The hydrodynamic diameters of these NCs were 92.1 ± 7.8 nm (CRDA), 115.3 ± 11.5 nm (CDA), and 69.8 ± 5.9 nm (HRDA), all exhibiting a surface negative charge of≈−22 mV (Figure 1B,C). Transmission electron microscopy (TEM) confirmed their uniform and spherical structure (Figure 1D; Figure S3, Supporting Information).

To demonstrate the applicability of our nanoformulation method, we encapsulated proteins with varying molecular weights (25–64 kDa) and isoelectric points (2.7–8.9) within the NCs (Figure S4, Supporting Information). The successful formation of NCs with diverse sizes and surface potentials (Figure 1E) and the efficient loading of these proteins (Table S1, Supporting Information) highlight the broad applicability of our synthetic strategy. To further investigate the versatility of the synthesis strategy, we explored the construction of different small molecules that share structural similarities with DA, possessing catechol and primary amine groups (Figure 1E; Figure S5, Supporting Information). Dynamic light scattering (DLS) analysis confirmed the nano‐structural integrity of these formulations. Consequently, this study provides a novel approach for the co‐delivery of small molecules with catechol and primary amine groups alongside macromolecules.

The stability of the NCs is crucial for their potential therapeutic application. We evaluated the stability of the NCs by monitoring changes in their hydrodynamic diameter over time in phosphate‐buffered saline (PBS). The NCs maintained stability in a neutral environment for up to 96 h, with no significant alterations in their size (Figure 1F). However, the NCs can only keep their stability for up to 48 h in cell medium and 24 h in FBS‐supplemented cell medium. (Figure S6, Supporting Information). Furthermore, the encapsulated CAT exhibited superior enzyme preservation compared to free CAT over the same duration at room temperature in PBS (Figure 1G), highlighting the protective role of the nanoformulation. To gain insights into the composition of the NCs, the concentrations of various components within the NCs were comprehensively quantified using a range of analytical techniques: DA levels were determined via high‐performance liquid chromatography (HPLC), protein content was assessed with the Bradford assay, boron (B) concentration was measured by inductively coupled plasma mass spectrometry (ICP‐MS), and the presence of cRGD was detected through its reaction with phenanthrenequinone (Figure S7, Supporting Information).^[^ ^23^ ^]^ The results demonstrated that the NCs maintained similar concentrations of DA and B, but exhibited varying levels of protein encapsulation (**Table** **1**). The DA/Protein ratio was 1.0 ± 0.2 for CRDA, 1.6 ± 0.4 for CDA, and 0.3 ± 0.02 for HRDA, with the lower ratio in HRDA attributed to the higher loading efficiency of HSA. The protein/cRGD ratio was 0.3 ± 0.09 for CRDA and 0.8 ± 0.04 for HRDA, confirming the successful incorporation of cRGD in the NCs.

**Table 1:** The concentration of different components within Nanocapsules.

	CRDA	CDA	HRDA
DA [µg/mg]	16.0 ± 3.3	19.5 ± 1.8	16.29 ± 2.5
Protein [µg/mg]	14.3 ± 2.9	14.0 ± 3.4	50.8 ± 4.3
B [µg/mg]	24.7 ± 4.0	22.5 ± 2.9	23.6 ± 5.5
cRGD [µg/mg]	42.7 ± 3.9	/	67.3 ± 7.2
DA/Protein	1.0 ± 0.2	1.6 ± 0.4	0.3 ± 0.02
Protein/cRGD	0.3 ± 0.09	/	0.8 ± 0.04

Previous research suggests that the pH of PD brain ranges from 6.4 to 6.6, accompanied by an elevated reactive oxygen species (ROS) level in the neuronal microenvironment.^[^ ^24^, ^25^, ^26^ ^]^ To investigate the controlled release of DA from the NCs under conditions mimicking the PD brain environment, we incubated the NCs in media with different pH values (7.4 and 6.4) and in the presence of elevated ROS (ROSUP) at pH 6.4. The released DA was verified using HPLC and mass spectrometry (Figure 1H,I). At pH 7.4, the release rate of DA from both CRDA and HRDA NCs was ≈10% over 72 h (Figures S9 and S10, Supporting Information). This release rate increased to 37.3% ± 5.7% for CRDA and 36.2% ± 3.8% for HRDA at a pH of 6.4 (Figures S11 and S12, Supporting Information). However, under the pH 6.4 + ROSUP condition, DA release from CRDA and HRDA decreased to 22.5% ± 2.3% and 12.9% ± 0.7%, respectively (Figures S13 and S14, Supporting Information). These results demonstrate that the mildly acidic conditions prompt DA release, whereas the presence of ROSUP leads to partial oxidation of DA (Figure 1J; Table S2, Supporting Information). The release rate of DA from CRDA was higher under the pH 6.4 + ROSUP condition compared to HRDA, indicating that CAT played a crucial role in reducing ROS levels and protecting DA from oxidation. The presence of CAT within the CRDA nanocomposite provided a protective effect against DA oxidation, as evidenced by a ≈15% reduction in release compared to the pH 6.4 condition without ROSUP. This finding suggests that the CRDA nanocomposite offers a significant degree of protection against DA oxidation, even under challenging oxidative stress conditions. Some studies indicated that the oxygen released from the reaction between CAT and ROSUP could promote DA oxidation;^[^ ^27^ ^]^ however, our findings indicate that this phenomenon is unable to occur at pH 6.4 (Figure S15, Supporting Information). This protective role of CAT highlights its potential significance in preserving the efficacy of DA in the oxidative environment of the PD brain.

### 2.2 Biocompatibility and BBB Penetration of Nanocapsules with In Vitro Model of PD

**Figure 2:**
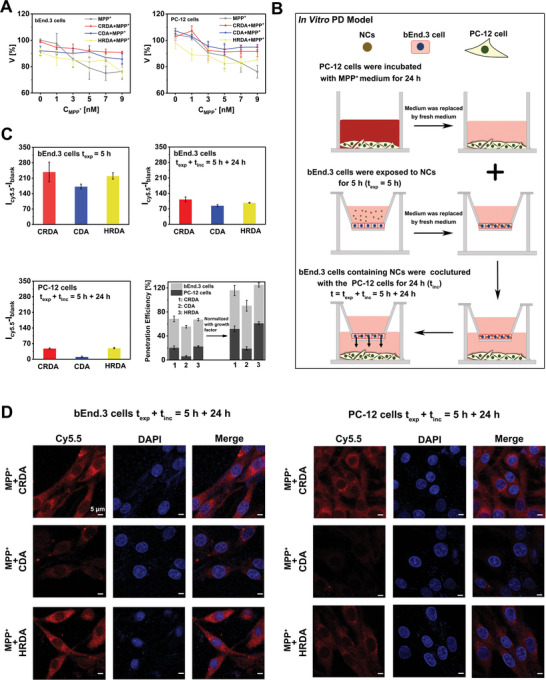
In Vitro PD Model and Penetration Efficiency of NCs. A) Cell viability (V) of PC‐12 cells after 24 h of treatment with various concentrations of MPP^+^ and NCs (C_DA_ = 50 µg mL^−1^, *n* = 3). B) Construction of an in vitro PD model using MPP^+^ incubation and a transwell system. PC‐12 cells were treated with MPP^+^ (3  mM) for 24 h, while bEnd.3 cells in the transwell filter were exposed to NCs for 5 h (t_exp_ = 5 h). Following this, the medium was refreshed after washing with PBS, and bEnd.3 cells were co‐incubated with the PC‐12 cells for an additional 24 h (t_inc_ = 24 h), totaling a combined incubation period of 29 h (t_exp_ + t_inc_ = 5 h + 24 h). C) Penetration efficiency [%] of NCs (C_DA_ = 50 µg mL^−1^) was evaluated by comparing the ratio of cellular Cy5.5 fluorescence (I_Cy5.5_) in bEnd.3 cells and PC‐12 cells at t_exp_ + t_inc_ = 5 h + 24 h relative to bEnd.3 cells at t_exp_ = 5 h, using flow cytometry (*n* = 3). D) Confocal microscopy images of NCs (C_DA_ = 50 µg mL^−1^) within the cells at t_exp_ + t_inc_ = 5 h + 24 h. The scale bar is 5 µm.

The biocompatibility of the NCs is a fundamental aspect of our study, as it establishes their suitability for further therapeutic evaluation. Initially, our investigation demonstrated the in vitro biocompatibility of NCs with mouse brain endothelial bEnd.3 cells and rat adrenal medullary carcinoma PC12 cells. This biocompatibility is a foundational aspect of our study, as it establishes the NCs' suitability for further therapeutic evaluation (Figure S16, Supporting Information).1‐Methyl‐4‐phenylpyridinium (MPP^+^), a neurotoxin used to simulate PD at the cellular level, was shown to increase oxidative stress, lead to mitochondrial dysfunction, and result in cell death.^[^ ^28^ ^]^ Remarkably, the NCs (C_DA_ = 50 µg mL^−1^) enhanced the viability of PC12 cells exposed to MPP^+^ across various concentrations (**Figure** **2**A). This treatment response was observed to be concentration‐dependent, with cell viability improvements being more pronounced at higher concentrations of MPP^+^.

Subsequently, LRP‐1 overexpressing bEnd.3 cells were utilized to assess the cellular uptake of Cy5.5‐labeled NCs. Flow cytometry analysis revealed that the uptake of NCs nearly reached saturation at 5 h post‐incubation (Figure S17, Supporting Information). Importantly, the presence of cRGD within CRDA and HRDA significantly improved the efficiency of cellular uptake compared to CDA, demonstrating that cRGD facilitates cell adhesion of NCs. To closely mimic the neuronal microenvironment of PD, we employed a transwell system in conjunction with MPP^+^ incubation to establish an in vitro PD model (Figure 2B). First, we incubated PC‐12 cells with MPP^+^ to replicate the neuronal toxicity observed in PD. PC‐12 cells were utilized as an in vitro model to assess the therapeutic effects of the nanodrugs due to their ability to synthesize DA and their susceptibility to neurotoxins. MPP^+^ exerts its toxicity by accumulating in mitochondria, where it blocks the activity of NADH‐ubiquinone oxidoreductase (complex I) of the mitochondrial electron transport system, leading to ATP depletion and leakage of DA into the cytosol from synaptic vesicles, which leads to ROS production.^[^ ^29^, ^30^ ^]^ Second, we employed a transwell system to mimic the BBB, where bEnd.3 cells were seeded on the apical side of the insert. The NCs were added to the apical compartment, allowing them to penetrate the bEnd.3 cell layer and reach the basolateral compartment containing the MPP^+^‐treated PC‐12 cells.

Therefore, the in vitro biofunction of NCs within PC‐12 cells incubated with MPP^+^ was analyzed after penetrating bEnd.3 cells. First, PC‐12 cells were incubated with 3 mM MPP^+^ for 24 h. bEnd.3 cells were exposed to Cy5.5‐labeled NCs for a duration of 5 h (t_exp_ = 5 h). Subsequently, the culture medium was refreshed for both bEnd.3 and PC‐12 cells. The bEnd.3 cells, now containing Cy5.5‐labeled NCs, were then co‐incubated with PC‐12 cells for an additional 24 h (t_inc_ = 24 h). The penetration efficiency of NCs into PC‐12 cells was quantitatively assessed through the analysis of cellular fluorescence both at t_exp_ = 5 h and the cumulative duration of t_exp_ + t_inc_ = 5 h + 24 h. Given that flow cytometry quantifies fluorescence at the single‐cell level, it is essential to adjust for cell growth to accurately determine penetration efficiency (Figure S18, Supporting Information). After normalization for growth factors, the penetration efficiency of CRDA and HRDA into PC‐12 cells was found to be 52.0% ± 4.6% and 61.3% ± 2.6%, respectively. In contrast, only 19.2% ± 2.9% of CDA‐modified NCs were able to traverse bEnd.3 cells and enter PC‐12 cells (Figure 2C). The confocal images visualized the difference in penetration efficiency between three NCs (Figure 2D). These findings demonstrate that incorporating the cRGD motif into NCs significantly enhances their ability to be internalized by cells and improves their penetration efficiency.

### 2.3 In Vitro Therapeutic Effects of Nanocapsules in PD model

**Figure 3:**
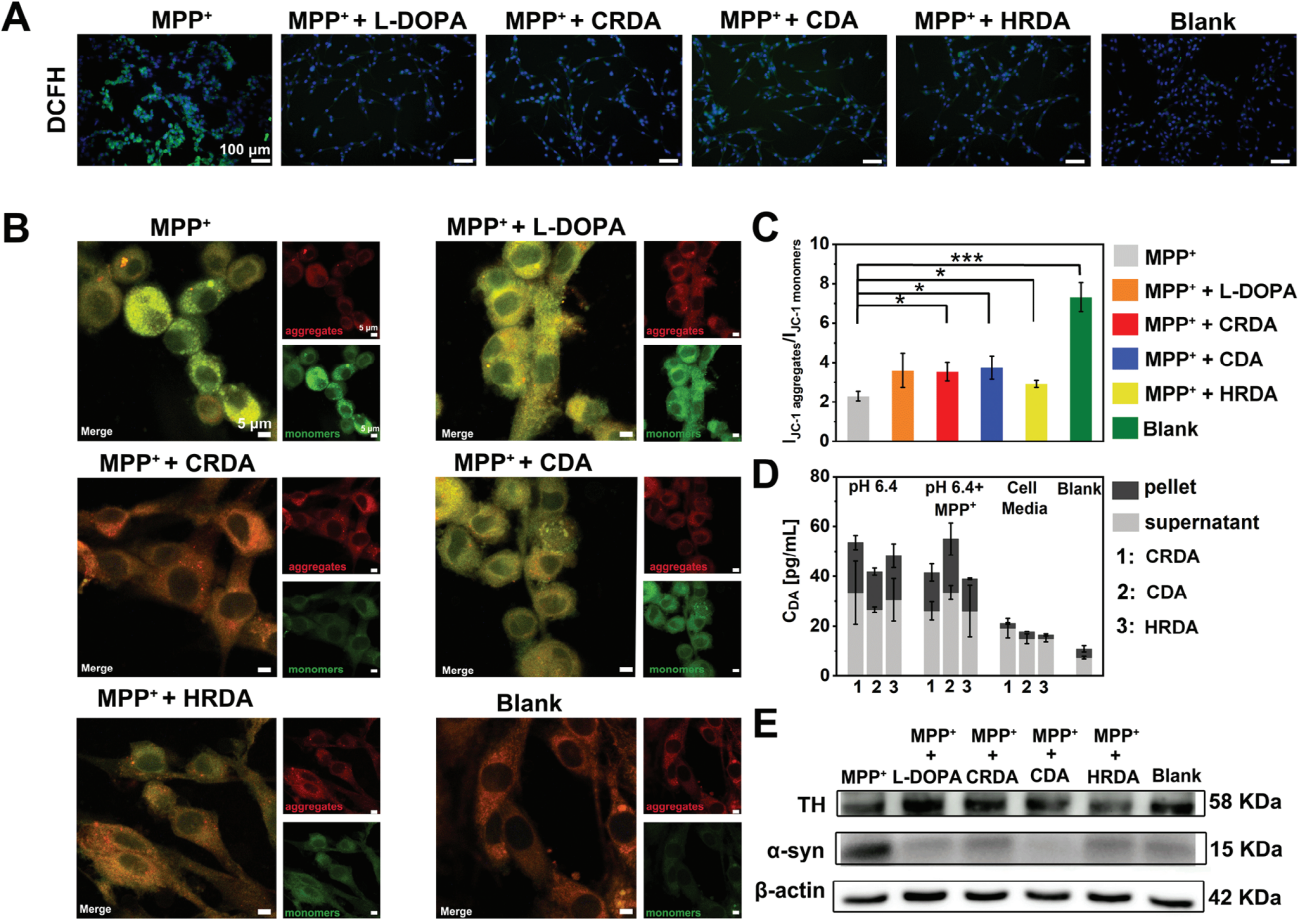
Therapeutic Effects of NCs with In Vitro PD Model. A) Measurement of DCFH fluorescence (I_DCFH_) within PC‐12 cells after various treatments at t_exp_ + t_inc_ = 5 h + 24 h. The scale bar is 100 µm. B) Confocal microscopy images and C) quantification of the ratios of JC‐1 aggregates to monomers in PC‐12 cells at t_exp_ + t_inc_ = 5 h + 24 h (*n* = 3). The scale bar is 5 µm. D) Release of DA from NCs (C_DA_ = 50 µg mL^−1^) into PC‐12 cells was measured after 5 h of incubation in complete cell media, pH 6.4 media, and pH 6.4 + ROSUP media (*n* = 3). E) Western blot images of TH and α‐syn in PC‐12 cells under various conditions at t_exp_ + t_inc_ = 5 h + 24 h. Statistical significance across different groups was determined using the one‐way ANOVA method, with significance levels denoted as *p < 0.05, **p < 0.01, ***p < 0.001.

The NCs were engineered to reduce neuroinflammation and enhance dopaminergic signaling following their penetration of the BBB. Subsequent in vitro analyses focused on the behavior of NCs after traversing bEnd.3 cell models. ROS levels and mitochondrial dysfunction are significant indicators of neuroinflammation. At t_exp_ + t_inc_ = 5 h + 24 h, fluorescence microscopy revealed a significant reduction in DCFH, a marker used to quantify ROS levels (**Figure** **3**A; Figure S19, Supporting Information). This reduction was markedly more pronounced in cells treated with L‐DOPA, CRDA, CDA and HRDA compared to those only treated with MPP^+^, indicating a superior capability of the former in mitigating oxidative stress. Mitochondrial dysfunction is a characteristic phenomenon associated with neuroinflammation. Furthermore, we assessed mitochondrial integrity through JC‐1 staining at t_exp_ + t_inc_ = 5 h + 24 h. JC‐1 produces the red fluorescence from aggregates in the healthy mitochondria but green fluorescence in monomeric form in the cells with damaged mitochondria. Therefore, the fluorescent intensity ratio of JC‐1 red/green can be used to quantify the maintenance of mitochondrial membrane potential. A decrease in the red/green ratio with MPP^+^ incubation of JC‐1 aggregates to monomers typically signifies the onset of cell death. Notably, our treatments counteracted this trend, as evidenced by an increased ratio, suggesting the protection of mitochondrial from ROS‐induced dysfunction (Figure 3B,C; Figure S20, Supporting Information). Next, we explored the controlled release of DA from NCs at the cellular level. The results indicated that a mildly acidic environment (pH 6.4) facilitated the degradation of NCs, subsequently releasing DA (Figure 3D). This controlled release of DA from NCs under conditions mimicking the PD brain environment highlights their potential to restore dopaminergic signaling and alleviate PD symptoms. Misfolded α‐syn is a hallmark of PD, while tyrosine hydroxylase (TH) plays a crucial role in DA biosynthesis.^[^ ^31^ ^]^ Therefore, their expression levels serve as significant indicators of dopaminergic signaling. At t_exp_ + t_inc_ = 5 h + 24 h, the expression of α‐syn and TH in PC‐12 cells was analyzed via Western blot. The treatments notably upregulated TH and downregulated α‐syn expression, suggesting a therapeutic effect against PD pathology (Figure 3E; Figure S21, Supporting Information). In summary, our findings demonstrate that the NCs, upon crossing the bEnd.3 cells, effectively reduce oxidative stress, mitigate mitochondrial dysfunction, and modulate the expression of critical enzymes associated with DA metabolism. Moreover, the capability of NCs to release DA under mildly acidic conditions further underscores their potential as a targeted therapeutic strategy for PD.

### 2.4 In Vivo Brain Targeting Ability of Nanocapsules

**Figure 4:**
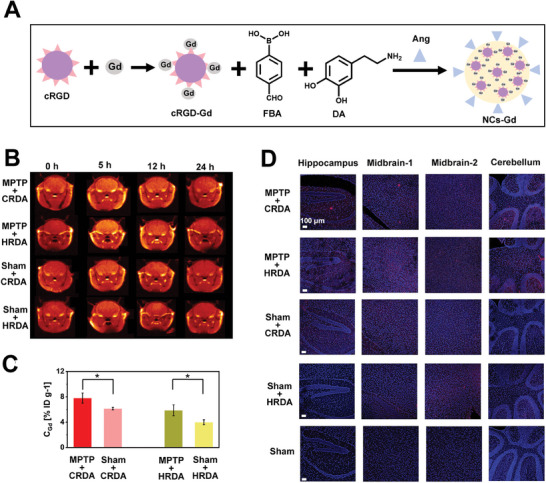
In Vivo Targeting Capability of NCs. A) Illustration of the synthesis process for Gd‐labeled NCs, outlining the step‐by‐step procedures involved in the preparation. B) MRI scans showcasing the brain of MPTP‐treated and sham mice following intravenous (i.v.) injection of CRDA and HRDA (C_DA_ = 600 µg kg^−1^) across various time intervals (*n* = 3). C) Quantification of Gd levels within the brain tissues of MPTP‐treated and sham mice post‐i.v. administration of CRDA and HRDA (C_DA_ = 600 µg kg^−1^) for 5 h. D) Confocal microscopy images of brain tissue sections from MPTP‐treated and sham mice after i.v. injection of CRDA and HRDA (C_DA_ = 600 µg kg^−1^) for 5 h. Statistical significance across different groups was determined using the one‐way ANOVA method, with significance levels denoted as *p < 0.05. The scale bar is 100 µm.

PD results in the upregulation of integrins within the BBB, which can be targeted by the cRGD peptide sequence. We hypothesized that NCs modified with cRGD could specifically target the PD‐altered BBB, enhancing the delivery of therapeutic agents to the affected brain regions. To validate our approach, we employed 1‐Methyl‐4‐phenyl‐1,2,3,6‐tetrahydropyridine (MPTP) to establish a PD model.^[^ ^32^ ^]^ MPTP serves as a classical neurotoxin that, upon crossing the BBB, is metabolized into its active form MPP^+^, within the brain. MPP^+^ selectively induces the loss of dopaminergic neurons in the substantia nigra, effectively replicating the neuropathological landscape of PD. To assess the brain‐targeting capabilities of our NCs, we initially administered Cy5.5‐labeled NCs to MPTP‐induced PD model mice and monitored the in vivo fluorescence using an in vivo imaging system (IVIS) (Figure S22, Supporting Information). The peak accumulation of Cy5.5‐labeled NCs in the brain was observed at 5 h post‐administration. To further investigate the brain‐targeting efficacy between sham and MPTP‐treated mice, we incorporated Gadolinium (Gd) into the NCs through interactions with the cRGD peptide to construct Gd labeled NCs (NCs‐Gd), enabling visualization by magnetic resonance imaging (MRI) (**Figure** **4**A; Figure S23, Supporting Information). MRI analysis confirmed that the accumulation of NCs‐Gd in the brain was discernible at 5 h post‐injection, with notably higher concentrations in MPTP mice compared to the sham group (Figure 4B; Figure S24, Supporting Information). Subsequent quantitative analysis was performed by collecting and digesting brain tissues at 5 h post‐injection for Inductively Coupled Plasma (ICP) measurement of Gd content. The findings revealed that Gd levels in the MPTP group were ≈1.4 times higher than those in the sham group, indicating a significant enhancement in brain accumulation within the disease model (Figure 4C; Figure S25, Supporting Information). Further investigations into the distribution of NCs within specific brain regions were conducted using frozen brain sections from mice administered with Cy5.5‐labeled NCs. Fluorescence imaging revealed that in MPTP mice, NCs were distributed across multiple brain areas, including the hippocampus, midbrain, and cerebellum, whereas in sham mice, NC distribution was primarily confined to the midbrain (Figure 4D). Although the incorporation of Gd into NCs altered their size distribution, zeta potential, and water solubility—which are factors known to influence in vivo distribution—these modifications did not preclude their utility as a comparative tool for assessing brain targeting efficacy between MPTP and sham groups. These findings collectively underscore the enhanced brain‐targeting capacity of the NCs, facilitated by the cRGD modification, which not only improved overall brain accumulation but also enabled penetration into a broader spectrum of brain regions.

### 2.5 Behavioral Evaluation of In Vivo Treatment with Nanocapsules

**Figure 5:**
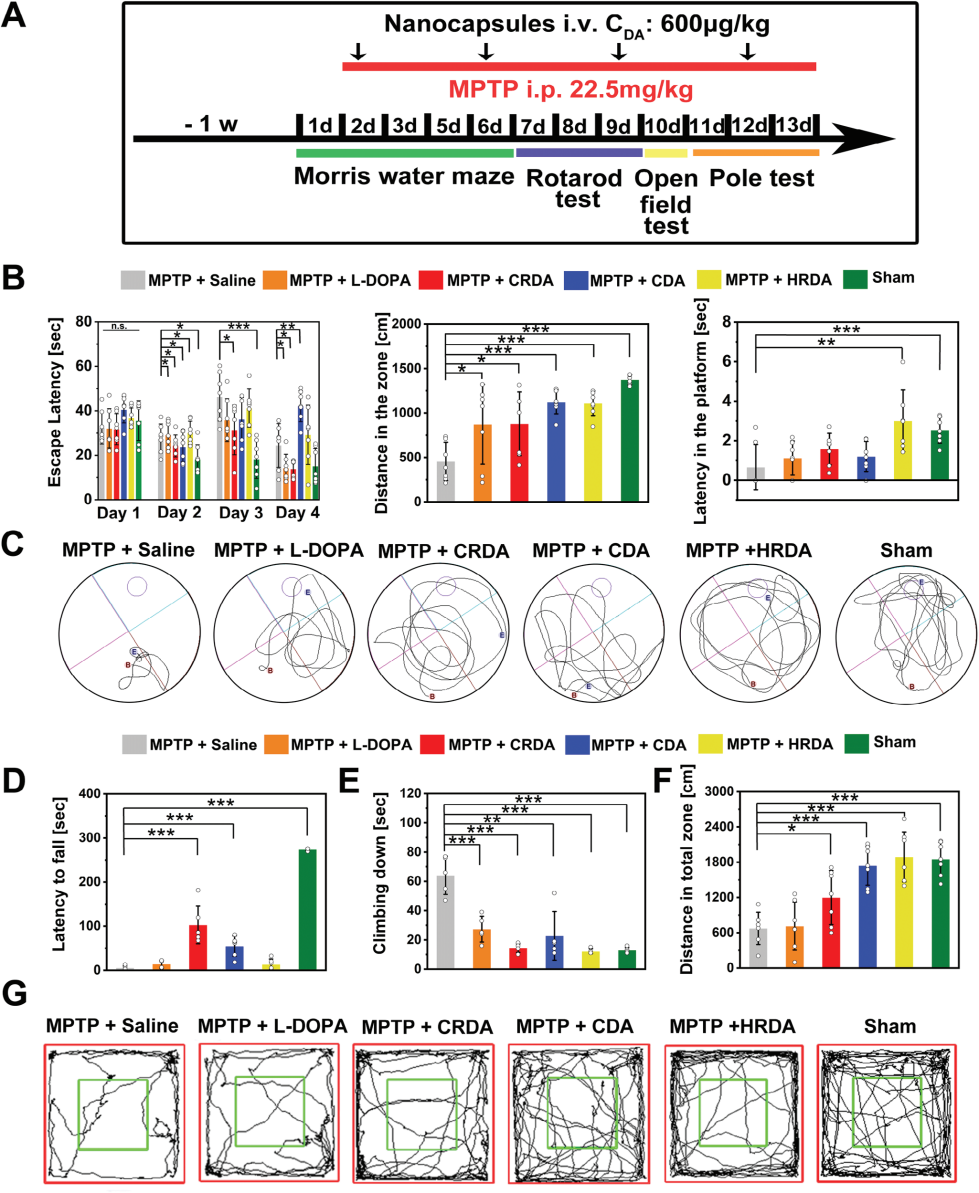
Therapeutic NCs Enhance Locomotor Activity, Spatial Learning, and Motor Coordination in PD Model Mice. A) Timeline depicting the schedule of behavioral tests conducted to assess the efficacy of therapeutic NCs on PD model mice. B) Learning curve of MPTP‐treated mice under various treatments over a 4‐day training period. Morris water maze test includes analyses of traveled distance to the target zone and latency to reach the platform on the testing day for all treatment groups (*n* = 7). C) Representative swimming paths in the Morris water maze test on the testing day. D) Latency to fall from the rotating rod in the rotarod test (*n* = 6). E) Climbing time (in seconds) recorded in the pole test (*n* = 5). F) Total distance traveled in the open field test (*n* = 7). G) Representative paths taken during the open field test for each treatment group. Statistical significance across different groups was determined using the one‐way ANOVA method, with significance levels denoted as *p < 0.05, **p < 0.01, ***p < 0.001.

To establish a PD model, MPTP was administered intraperitoneally at a dose of 22.5 mg kg^−1^ daily for 13 days to mice, as MPTP is known to selectively destroy dopaminergic neurons in the substantia nigra. Concurrently, NCs were administered to these mice via tail vein injection every three days to evaluate their therapeutic potential against PD (**Figure** **5**A). The mice were organized into six groups for behavioral assessment: 1) MPTP‐treated mice receiving saline (MPTP + saline), 2) MPTP‐treated mice receiving L‐DOPA (MPTP + L‐DOPA), 3) MPTP‐treated mice receiving CRDA (MPTP + CRDA), 4) MPTP‐treated mice receiving CDA (MPTP + CDA), 5) MPTP‐treated mice receiving HRDA (MPTP + HRDA), and 6) sham group. Given that PD symptoms encompass both non‐motor symptoms, such as cognitive impairment, and motor symptoms, our evaluations began with spatial learning and memory assessments using the Morris water maze—a benchmark test for rodent behavior (Figure 5B). The escape latency for the sham group diminished progressively across 4 days of trials, indicative of learning. Conversely, the MPTP + saline group showed no significant change in escape latency, underscoring locomotor impairment and delayed platform finding. Unlike the MPTP+ L‐DOPA and CRDA group, which demonstrated a decrease in escape latency over the 4‐day training session, the MPTP + CDA and MPTP + HRDA groups did not show a reduction in escape latency. After treatment, all groups showed improvements in locomotion and navigational efficiency during the maze testing session, although the extent of these improvements varied. Notably, the MPTP + CDA and MPTP + HRDA treatment groups had significantly longer distances traveled and greater platform latency compared to the MPTP + L‐DOPA and MPTP + CRDA groups (Figure 5C). Interestingly, the mice in the CDA and HRDA treatment groups exhibited inconsistent performance throughout the learning process. Motor coordination and balance were further assessed using the rotarod test (Figure 5D). After two days of training, the sham group completed the test within 300 s, while the MPTP + saline group exhibited a significant reduction in fall latency. CRDA treatment notably improved fall latency, indicating enhanced balance and motor coordination in MPTP‐treated mice. The pole test, used to evaluate movement disorders resulting from striatal DA loss, showed that the MPTP + saline group took longer to descend, reflecting movement slowness. All treatments ameliorated movement speed and reduced descent time (Figure 5E). Lastly, the open field test assessed spontaneous locomotor activity and anxiety levels. The MPTP treatment significantly diminished overall distance moved, center distance (indicated by green box), and center time, indicating reduced locomotor activity, exploratory behavior, and increased anxiety in the MPTP + saline group (Figure 5F,G; Figure S26, Supporting Information). Treatment with the three NCs improved general locomotion and exploratory behavior. Body weight changes in mice can serve as an additional metric for assessing therapeutic efficacy. Treatment with MPTP significantly reduced body weight, whereas subsequent administration of CRDA was associated with an increase in body weight in MPTP‐treated mice (Figure S27, Supporting Information). Although the various behavioral tests assessed different aspects of therapeutic outcomes, CRDA demonstrated superior efficacy through a stable increase in learning and memory, enhanced locomotor activity, and improved motor coordination. In summary, CRDA showed the most promising therapeutic outcomes based on behavioral tests, highlighting its potential as a synergistic therapy for PD.

### 2.6 Therapeutic Nanocapsules Enhance the Dopaminergic Signaling

**Figure 6:**
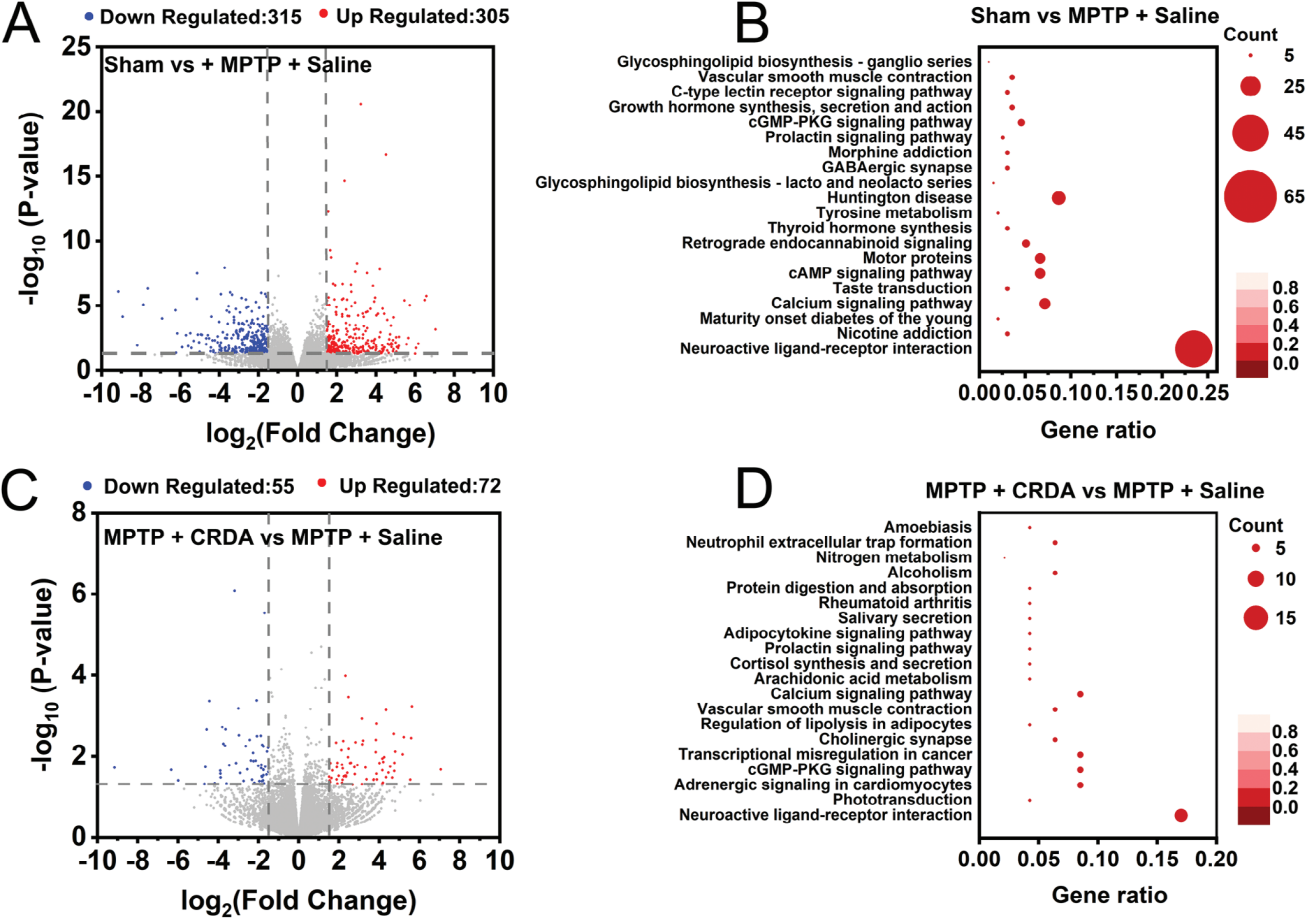
mRNA Sequencing Unveils Gene‐Level Mechanisms. A) Volcano plot illustrating gene expression differences between Sham and MPTP + Saline cases, with 315 upregulated genes in red and 305 downregulated genes in green. B) The top 20 KEGG pathways associated with differentially expressed genes (DEGs) in the Sham vs MPTP + Saline comparison. C) Volcano plot for the comparison between MPTP + CRDA and MPTP + Saline cases, showing 72 upregulated genes in red and 55 downregulated genes in green. D) The top 20 KEGG pathways for DEGs in the MPTP + CRDA vs MPTP + Saline comparison. These results are from three independent samples (*n* = 3).

To elucidate the therapeutic effects of NCs, we employed mRNA sequencing to analyze gene expression changes within the substantia nigra. Differential expression analysis, visualized through a volcano plot with a cutoff of *P* ≤ 0.05 and a fold change (FC) ≥ 1.5, revealed 315 genes significantly downregulated and 305 genes significantly upregulated between Sham and MPTP + Saline groups (**Figure** **6**A). Kyoto Encyclopedia of Genes and Genomes (KEGG) pathway enrichment analysis of these genes highlighted the neuroactive ligand‐receptor interaction pathway as the most enriched (Figure 6B). Applying the same methodology to compare MPTP + CRDA and MPTP + Saline groups, we identified 55 genes with significant downregulation and 72 genes with significant upregulation within the neuroactive ligand‐receptor interaction pathway (Figure 6C,D). This pathway is integral to neurotransmitter signaling, neural activity, learning, and memory, underscoring the potential of NCs to regulate dopaminergic neurotransmission.^[^ ^33^, ^34^ ^]^

Our NCs are specifically designed to enhance DA delivery to the brain parenchyma and amplify DA‐related signaling. To further investigate NCs' impact on dopaminergic signaling, we systematically measured related proteins. TH, the rate‐limiting enzyme in DA synthesis, catalyzes the conversion of tyrosine to L‐DOPA and subsequently to DA.^[^ ^35^ ^]^ The dopamine transporter (DAT) plays a pivotal role in DA reuptake into presynaptic terminals, essential for maintaining DA concentration and availability.^[^ ^36^ ^]^ DA operates through various receptors, with the dopamine D2 receptor (DRD2) located in key areas such as the striatum, substantia nigra, and hippocampus, regulating DA synthesis and TH activity.^[^ ^37^ ^]^ DRD2 also influences neuronal excitability and signal transduction, affecting behavior and cognitive functions. Additionally, brain‐derived neurotrophic factor (BDNF) promotes the survival and differentiation of dopaminergic neurons, playing a significant role in DA synthesis and release.^[^ ^38^ ^]^ These proteins are closely related to dopaminergic signaling from synthesis to function was systematically studied.^[^ ^39^ ^]^

**Figure 7:**
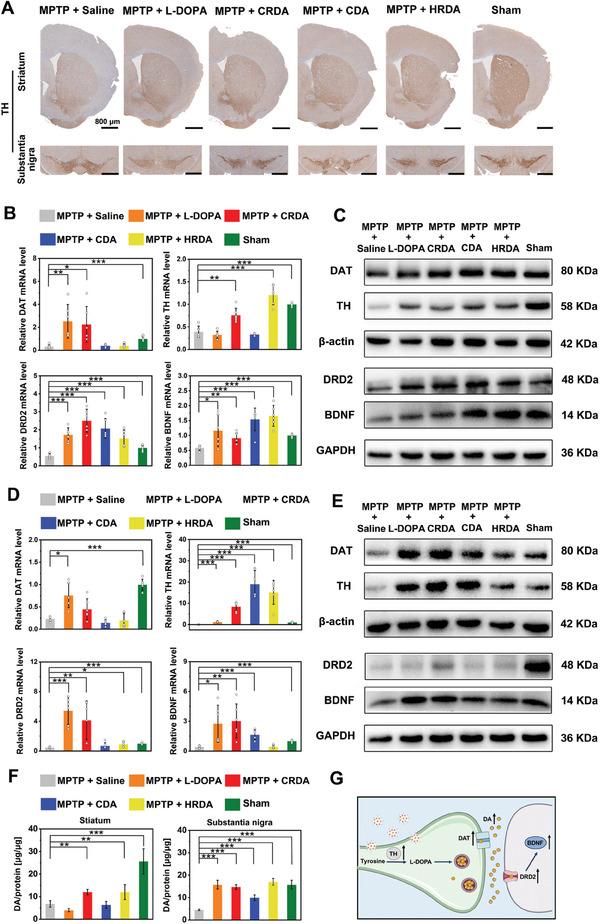
Enhancement of Dopaminergic Signaling by Therapeutic NCs at Gene and Protein Levels. A) IHC for TH in brain sections of the striatum and substantia nigra across all experimental groups. The scale bar is 800 µm. B) Analysis of relative mRNA expression levels for TH, DAT, DRD2, and BDNF in the striatum of mice from each group (*n* = 6), conducted via RT‐qPCR. C) Western blot analysis revealing protein expression of TH, DAT, DRD2, and BDNF in the striatum of MPTP‐treated mice receiving various NC treatments. D) RT‐qPCR analysis of relative mRNA levels for TH, DAT, DRD2, and BDNF in the substantia nigra of mice across all groups (*n* = 6). E) Protein expression of TH, DAT, DRD2, and BDNF in the substantia nigra of MPTP‐treated mice under different treatment conditions, as shown by western blot analysis. Other two replicates were shown in Figures S29 and S30 (Supporting Information). F) Measurement of DA levels in the striatum and substantia nigra of MPTP mice subjected to various treatments (*n* = 4). G) A summary statement that therapeutic NCs significantly boost DA‐related proteins from synthesis to functionality, affirming their effect at both mRNA and protein levels. Statistical significance across different groups was determined using the one‐way ANOVA method, with significance levels denoted as *p < 0.05, **p < 0.01, ***p < 0.001.

Immunohistochemistry (IHC) images of TH demonstrated a significant loss in the striatum and substantia nigra of MPTP‐treated mice, which was ameliorated following treatment, with NCs showing superior therapeutic outcomes compared to L‐DOPA (**Figure** **7**A; Figure S28, Supporting Information). Reverse transcription‐quantitative polymerase chain reaction PCR (RT‐qPCR) and western blot analyses systematically evaluated TH, DAT, DRD2, and BDNF across all groups in the striatum and substantia nigra, showing a reduction in these proteins at both mRNA and protein levels following MPTP administration. All treatments notably upregulated these four proteins in MPTP‐treated mice, with CRDA exhibiting the most significant therapeutic effects (Figure 7B–E; Figures S29–S31, Supporting Information). Additionally, the enhancement of dopaminergic signaling‐related proteins is more pronounced in the substantia nigra as compared to striatum (Figure 7; Figure S31, Supporting Information). Furthermore, direct measurement of DA levels in the striatum and substantia nigra via ELISA indicated that MPTP administration substantially reduced dopamine levels. CRDA and HRDA treatments elevated DA levels in the striatum and substantia nigra of MPTP‐treated mice by ≈1.8‐ and 3.5‐fold, respectively. These increases in DA levels were more pronounced compared to those observed with L‐DOPA and CDA treatments. Furthermore, the results indicated that the increase in DA levels was more significant in the substantia nigra than in the striatum (Figure 7F).

In summary, mRNA sequencing suggested that NCs modulate the neurotransmitter pathway. A comprehensive analysis of DA‐related proteins, from synthesis to function, at both mRNA and protein levels, revealed that NCs not only increase DA levels but also upregulate associated proteins, highlighting their potential in therapeutic applications (Figure 7G). Treatments with CRDA and HRDA demonstrated more pronounced improvements in the expression of dopaminergic signaling‐related proteins and DA levels compared to L‐DOPA and CDA. These findings highlight the advantages of nanoformulation and cRGD modification in enhancing the therapeutic efficacy of the nanodrugs. Furthermore, the improvements observed in the substantia nigra were more significant than those in the striatum.

### 2.7 Decrease in Neuroinflammation through the Reduction of Endoplasmic Reticulum Stress

**Figure 8:**
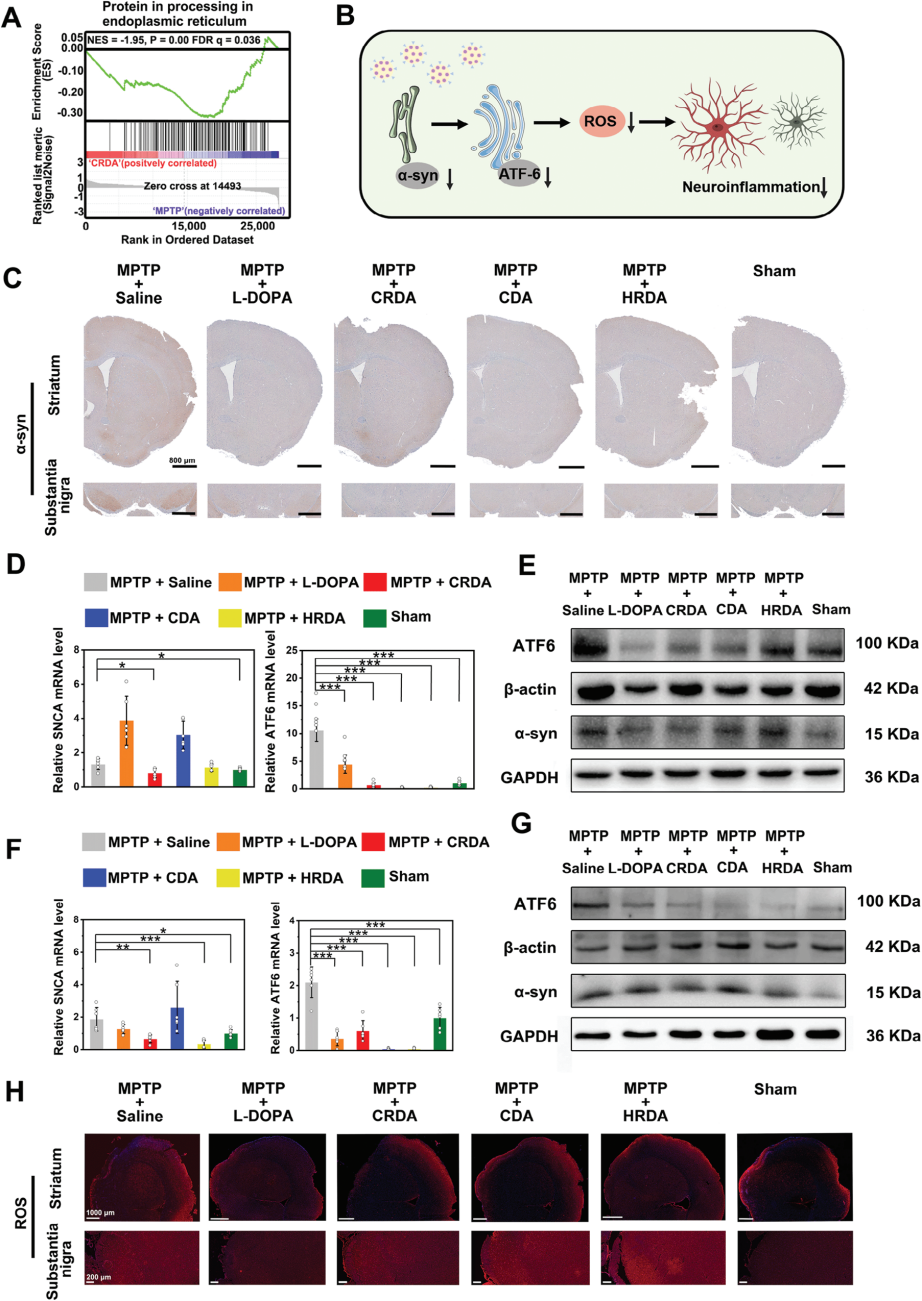
Impact of Therapeutic NCs on Neuroinflammation via ER Stress Modulation. A) GSEA of KEGG terms comparing MPTP + CRDA to MPTP + Saline groups. B) The mechanism of reduction in neuroinflammation of NCs by decreasing α‐syn, ATF6, and ROS. C) IHC for α‐syn in brain sections of the striatum and substantia nigra for all experimental groups. The scale bar is 800 µm. D,F) RT‐qPCR analysis of relative mRNA levels for α‐syn and ATF6 in the striatum D) and substantia nigra F) of mice, quantifying gene expression across groups (*n* = 6). E,G) Western blot analysis for protein expression of α‐syn and ATF6 in the striatum E) and substantia nigra G) of mice. Other two replicates were shown in Figures S29 and S30 (Supporting Information). H) ROS staining in brain sections of the striatum and substantia nigra across all groups. The scale bar is 1000 and 200 µm. Statistical significance across different groups was determined using the one‐way ANOVA method, with significance levels denoted as *p < 0.05, **p < 0.01, ***p < 0.001.

ER stress has been implicated in the pathogenesis of PD, contributing to neuroinflammation. The results of Gene Set Enrichment Analysis (GSEA) from mRNA sequencing demonstrated that CRDA significantly downregulated the protein processing pathway in the ER, as evidenced by a Normalized Enrichment Score (NES) of ‐1.95, a P‐value of 0.00, and a False Discovery Rate (FDR) q‐value of 0.036 (**Figure** **8**A). The ER plays a crucial role in protein synthesis, and misfolded or unfolded α‐syn has been shown to contribute to sustained ER stress, triggering the activation of the unfolded protein response (UPR).^[^ ^4^ ^]^ The UPR is mediated by complex stress sensors located at the ER membrane, among which ATF6 is a critical sensor associated with UPR activation and directly related to inflammatory responses, including an increase in ROS and activated glial cells (Figure 8B).^[^ ^40^ ^]^

To systematically investigate the effects of NCs on ER stress, we measured the expression levels of α‐syn, ATF6, ROS, and neuroinflammation markers. IHC staining of α‐syn revealed that the MPTP + saline group exhibited high intensity in both the striatum and substantia nigra, whereas all treatments significantly reduced α‐syn concentration in these areas (Figure 8C; S29–S32 only with Figure S32, Supporting Information). Subsequent RT‐qPCR and Western blot analyses confirmed that MPTP administration upregulated α‐syn and ATF6 at both mRNA and protein levels in striatum and substantia nigra, while all treatments downregulated these markers (Figure 8D–G; Figure S29–S31, Supporting Information). Among the experimental results, the reduction of α‐syn and ATF6 in the groups treated with CRDA is more pronounced than in the group treated with other treatments. This result aligns with dopaminergic signaling and the reduction is particularly noticeable in the substantia nigra. Additionally, ROS staining indicated a decrease in ROS intensity in the striatum and substantia nigra of MPTP‐treated mice following therapeutic interventions (Figure 8H; Figure S33, Supporting Information). These findings suggest that NCs effectively reduce ER stress by downregulating α‐syn and ATF6 expression, consequently decreasing neuroinflammation, as evidenced by the reduction in ROS. The ability of NCs to modulate the ER stress pathway and alleviate neuroinflammation highlights their potential as a therapeutic strategy for PD.

**Figure 9:**
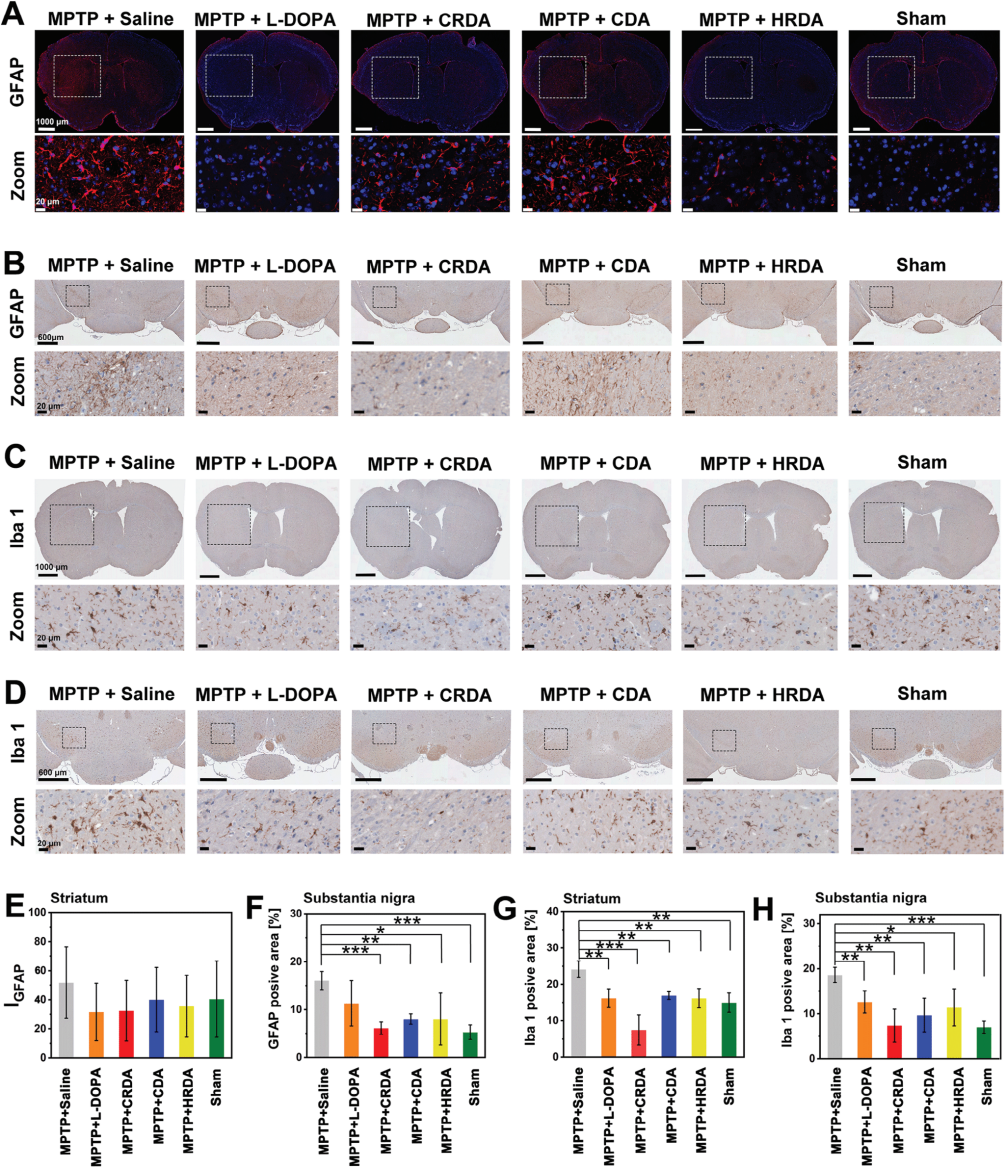
Reduction of Neuroinflammation by Therapeutic Nanocapsules. A) Immunofluorescence staining for GFAP in the striatum of mice from all experimental groups. B) IHC staining for GFAP in the substantia nigra across all groups. C,D) IHC staining for ‐ Iba‐1 in the striatum C) and substantia nigra D) for each group. E–H) Quantitative analysis of GFAP and Iba 1 expression in the striatum and substantia nigra, performed using Image J software. The presented quantitative results of IHC are derived from three independent samples (*n* = 3). Statistical significance across different groups was determined using the one‐way ANOVA method, with significance levels denoted as *p < 0.05, **p < 0.01, ***p < 0.001.

Astrocytes and microglia play crucial roles in neuroinflammation, a key feature of PD. Glial fibrillary acidic protein (GFAP), predominantly expressed in astrocytes, serves as a marker of astrocyte activation in response to neuroinflammation.^[^ ^41^ ^]^ GFAP staining revealed that MPTP administration significantly increased GFAP levels in the striatum and substantia nigra, which was reversed by the treatments (**Figure** **9**A,B,E,F). Among these, CRDA and HRDA treatments demonstrated superior therapeutic outcomes. Similarly, ionized calcium‐binding adaptor molecule 1 (Iba‐1), a biomarker for microglial activation and a key feature of neuroinflammation,^[^ ^42^ ^]^ was also evaluated. IHC staining showed that MPTP treatment significantly increased Iba‐1 intensity in the striatum and substantia nigra, while treatments, particularly with CRDA, reduced Iba‐1 levels (Figure 9C,D,G,H). The cyclical process of DA loss and neuroinflammation accelerates PD progression. To address this, our NCs are engineered to deliver CAT into the brain parenchyma, aiming to mitigate neuroinflammation and enhance DA efficacy. Collectively, our findings indicate that CRDA alleviate ER stress by reducing α‐syn, ATF6, and ROS levels, thereby diminishing neuroinflammation. Integrating the data on dopaminergic signaling, the in vivo therapeutic outcomes of CRDA were more pronounced than those of other treatments. This finding underscores the importance of a synergistic approach in the treatment of PD, where the combination of nanoformulation, cRGD modification, and the CAT loading work together to enhance the overall therapeutic efficacy.

In addition to their therapeutic efficacy, the in vivo biocompatibility of the NCs was confirmed following the treatment regimen. Serum biomarkers associated with liver and kidney function, including alanine aminotransferase (ALT), aspartate aminotransferase (AST), blood urea nitrogen (BUN), and creatinine (CREA), showed no significant changes in most groups, with only a marginal decrease in CREA levels in the MPTP + HRDA group compared to the Sham group (Figure S34A–D, Supporting Information). Additionally, hematoxylin and eosin (HE) staining of vital organs such as the heart, liver, spleen, lung, and kidney revealed no apparent tissue damage post‐NCs treatments (Figure S34E, Supporting Information). Collectively, these findings underscore the favorable in vivo biocompatibility of the NCs, highlighting their potential as therapeutic agents.

## 3 Conclusion

This study aimed to address the challenges associated with the delivery of therapeutic agents to the brain for the treatment of PD. We introduced a novel approach for the co‐delivery of CAT and DA directly to the brain parenchyma using carrier‐free NCs. The carrier‐free NCs constructed by reaction with DA and FBA, which serve as a versatile platform for the simultaneous delivery of macromolecules and small molecules containing primary amine and catechol groups. The stability of the NCs under neutral conditions and their controlled degradation in mildly acidic environments enable the efficient release of DA at the target site. The incorporation of cRGD modification within CAT enhances the delivery efficiency of the NCs to the parkinsonian brain by ≈1.4‐fold, as it enables the recognition of integrins overexpressed on the BBB in the PD state. This targeted delivery approach also allows for a broader distribution of the therapeutic agents within brain tissues.

The therapeutic strategy employed by carrier‐free NCs offers a synergistic approach to PD treatment. First, the NCs significantly increase brain DA levels by 1.8‐fold in the striatum and 3.5‐fold in the substantia nigra, respectively. The NCs further boost the activity of dopaminergic signaling‐related proteins, substantially alleviating PD symptoms. Second, the NCs markedly reduce neuroinflammation, which not only slows the progression of PD but also protects DA from oxidation. By integrating symptomatic and etiological treatments, our NCs present a comprehensive and innovative approach to PD therapy. This study demonstrates the potential of biocompatible, multi‐target nanoformulations in overcoming the limitations of current PD treatments.

In conclusion, our findings provide a new strategic avenue for advancing the treatment of PD and pave the way for future research and clinical applications of targeted, synergistic nanotherapeutics in the field of neurodegenerative diseases.

## 4 Experimental Section

### Synthesis and Characterizations of CAT‐cRGD

In order to make the CAT‐cRGD complex, first, 30 mg of CAT was dissolved in 10 mL of PBS to create a solution. Then, 182.25 µL of N‐Succinimidyl iodoacetate (SIA; 1 mg mL^−1^ in dimethylformamide (DMF); Aladdin, #N133327, China) was slowly added to the CAT solution. This mixture was then maintained at room temperature (RT) in a dark environment to enable the reaction to proceed for 1.5 h. Following the initial reaction period, the CAT‐SIA conjugate was isolated using an ultracentrifuge filter with a molecular weight cutoff of 100 kDa. The centrifugation was conducted at 6000 rpm for a duration of 30 min. Concurrently, cRGD, sourced from GL Biochem, were dissolved in a mixture of acetonitrile and ultrapure water at a ratio of 1:4 to achieve a concentration of 1 mg mL^−1^. This solution was combined with 461 µL of the prepared cRGD solution and 49.5 µL of hydroxylamine (Aladdin, #H164487, China) and then added to the CAT‐SIA solution at a temperature of 4 °C. The resulting solution was allowed to react overnight. Following the overnight incubation, 77.63 µg of cysteine (Aladdin, #C100539, China) was introduced into the reaction mixture. To purify the resultant CAT‐cRGD product, the solution underwent dialysis against a 100 kDa cutoff membrane at 4 °C for 6 h. The molecular weight of the purified CAT‐cRGD conjugate was determined using 10% sodium dodecyl sulfate‐polyacrylamide gel electrophoresis (SDS‐PAGE). The enzymatic activity of the CAT‐cRGD complex was assessed via a colorimetric assay. This involved a reaction mix containing 3,3′,5,5′‐tetramethylbenzidine (C_TMB_ = 2.5 mm, Aladdin, #T100415, China), hydrogen peroxide (C_H2O2_ = 88.2 mM, Aladdin, #H433857, China), and the CAT‐cRGD or CAT alone (both at a concentration of 1 mg mL^−1^) in sodium acetate‐acetic acid (NaOAc‐HAc) buffer with a pH of 5. The progression of the reaction was monitored and documented using a digital camera. Using the same procedure, a human serum albumin (HSA)‐cRGD conjugate was also synthesized for comparison. The key point of this procedure was that the reaction and purification steps should be carried out at 4 °C. Despite these precautions, a portion of the CAT may lose its enzymatic viability after the cRGD modification. Therefore, it was essential to quantify the CAT‐cRGD by measuring the enzymatic viability of CAT rather than relying solely on the concentration.

### Synthesis and Characterization of Carrier‐Free Nanocapsules

An inner‐core nanoparticle solution was synthesized by dissolving a mixture of CAT‐cRGD/CAT/HSA‐cRGD (4 mg), DA (m_DA_ = 3.8 mg, Aladdin, # A303863, China), and FBA (m_FBA_ = 1.5 mg, Aladdin, # F106771, China) in 10 mL of Citric acid–Na_2_HPO_4_ buffer (pH 7.8) containing ascorbic acid (AA, m_AA_ = 8.8 mg, Aladdin, # A103534, China). The reaction was allowed to proceed for 3.5 h at RT. The nanoparticles were then purified using a centrifuge at 1500 rpm for 15 min, followed by dialysis against ultrapure water at pH 8.0 over 6 h using a 100 kDa membrane. To demonstrate the versatility of the synthesis procedure, the macromolecules were replaced with pepsin (Pep, Aladdin, # P110927, China), bovine serum albumin (BSA, Aladdin, # A104912, China), hemoglobin (Hb, Aladdin, #H195714, China), and α‐chymotrypsin (α‐CT, Aladdin, # C128654, China). Similar methods were applied to small molecules such as levodopa (L‐DOPA, Aladdin, #D111049, China) and norepinephrine (NE, Aladdin, #L335487, China).

Subsequently, these inner‐core nanoparticles were incubated with Ang supplied by Shanghai GL Biochem at a mass ratio of 1:0.5 for 1 h at RT. The therapeutic nanoparticles formed were further purified via dialysis in ultrapure water at pH 8.0 for 6 h using a 100 kDa membrane, followed by lyophilization to yield the final NCs. The critical aspect of the carrier‐free NCs synthesis was the inclusion of an anti‐oxidant reagent, such as ascorbic acid, in the buffer. DA, which contains two phenol groups, is highly susceptible to oxidation. To construct the NCs effectively, it was crucial to use non‐oxidized dopamine in the reaction.

The hydrodynamic size distributions (N(d_h_)) of the obtained NCs were characterized using DLS in a UV‐cuvette (ZH 8.5 mm; Deckel; Sarstedt, Germany). The ζ‐potential distribution (I(ζ)) was also recorded using DLS with laser Doppler anemometry (LDA). To assess the stability of the NCs, they were incubated in PBS for 96 h and characterized periodically using DLS. The morphology of three representative NCs was imaged using TEM (Talos F200S SUPERX, Thermo Scientific, USA).

The enzyme stability of CAT within the CRDA and CDA was evaluated in PBS for 96 h. CRDA, CDA, and free CAT were each dissolved in 1 mL of PBS (C_CAT_ = 50 µg mL^−1^) and maintained at RT. CAT activity was first measured at t = 0 h as a control using a testing kit (Solarbio, #BC0200, China), with subsequent CAT viability normalized to 100% at t = 0 h and expressed as percentage viabilities at different time points. Details on the quantification of components within the NCs are provided in the Supporting Information.

### Stimuli‐Responsive Release of DA

To investigate the stimuli‐responsive release of DA, HPLC was initially employed in conjunction with Electrospray Ionization Mass Spectrometry (ESI‐MS) for verification purposes. CRDA and HRDA were prepared at a concentration of C_DA_ = 30 µg mL^−1^. These samples were incubated in citric acid‐Na_2_HPO_4_ buffer solutions at different pH levels (7.4, 6.4) and also in a citric acid‐Na_2_HPO_4_ buffer (pH 6.4) supplemented with ROUSP (ratio 1:1000, Beyotime, #S0033S, China) for a duration of 72 h. The released DA was isolated using an ultracentrifuge filter (5 kDa) at 8000 rpm for 30 min. The filtrates were collected and their DA content was quantified using HPLC. The HPLC procedure, as outlined in Supporting Information, was consistently applied across all samples. Fractions with retention times ranging from 2 to 2.5 min were gathered and subsequently analyzed by ESI‐MS to verify the molecular weight of DA, thereby confirming its stimuli‐responsive release. Additionally, the DA release profiles of CRDA and HRDA at an elevated concentration (C_DA_ = 50 µg mL^−1^) were quantitatively assessed through HPLC. The HPLC analysis and the preparation of the standard curve were conducted according to the methods described in Supporting Information.

Furthermore, the influence of pH on the stimuli‐responsive release of DA was visually documented. During a 2 h incubation period, CAT (C_CAT_ = 1 mg mL^−1^) and DA (C_DA_ = 1 mg mL^−1^) were incubated in citric acid‐Na_2_HPO_4_ buffer solutions at pH 7.4 and 6.4, with and without the addition of ROUSP. After incubation, the effects of pH were visually documented through photographs taken with a digital camera.

### Cell Cultures, Cytotoxicity Assays, and Cellular Uptake

The cell experiments of study adopted mouse brain endothelial cell line bEnd.3 (passage numbers 5–15) and the rat pheochromocytoma cell line PC‐12 (passage numbers 5–15). These cell lines were grown in Dulbecco's Modified Eagle Medium (DMEM, Gibco, USA) with 10% fetal bovine serum (FBS) (Excell, China) and 100 U mL^−1^ penicillin/streptomycin (Gibco, USA), and were incubated at 37 °C in a 5% CO_2_ atmosphere. To assess the cytotoxic effects of CRDA, CDA, and HRDA on bEnd.3 and PC‐12 cells, the resazurin assay was employed for cell viability evaluation, which is described in the Supporting Information. The methods of time‐dependent cellular uptake are provided in Supporting Information.

### In Vitro PD Model for Detecting NC Penetration Efficiency

To simulate the neuronal microenvironment affected by PD, an in vitro model was established using PC‐12 cells and bEnd.3 cells. PC‐12 cells were plated at 100,000 cells per well in 24‐well plates (1.9 cm^2^ surface area) and cultured in 1 mL of complete medium. On the next day, these cells were treated with MPP^+^ at a concentration of 3 mM Mfor 24 h to induce a PD‐like condition. Concurrently, bEnd.3 cells were seeded on transwell inserts (pore size: 0.4 µm, diameter: 6.5 mm, Costar, USA) at a density of 60,000 cells per well. The following day, these endothelial cells were exposed to the NCs (C_DA_ = 50 µg mL^−1^) in 0.3 mL of complete medium for 5 h (t_exp_ = 5 h). Part of this setup was designated for flow cytometry analysis to assess cellular uptake, while the remaining setup was reserved for further examination. Post‐exposure, both PC‐12 and bEnd.3 cells were washed with PBS and then incubated in a fresh medium. Subsequently, the bEnd.3 cell‐containing inserts were placed over the PC‐12 cells treated with MPP^+^ for an additional incubation time for 24 h to evaluate NC penetration from the apical to the basolateral chamber (t_inc_ = 24 h). The intracellular fluorescence in cells from both chambers was quantified via flow cytometry, considering both the exposure and incubation periods (t_exp_ + t_inc_ = 5 h + 24 h), to ascertain the penetration efficiency. This assessment accounted for cell proliferation, adjusting the penetration efficiency calculations based on cell counts and growth factors as detailed in Figure S18 (Supporting Information). Additionally, confocal laser scanning microscopy (CLSM; LSM900, Zeiss, Germany) was employed for visualizing the penetration efficiency. At the conclusion of the experiment, cells were fixed with 4% paraformaldehyde, rinsed, and stained with DAPI (Beyotime, #C1006, China) before imaging under CLSM

### Therapeutic Outcomes of NCs Characterized by an In Vitro PD Model

To evaluate the therapeutic potential of NCs in a PD model, their anti‐inflammatory capabilities were focused on, specifically their effectiveness in scavenging ROS and regulating mitochondrial dysfunction. The intracellular ROS levels were determined using 2',7' –dichlorofluorescein diacetate (DCFH‐DA), a widely accepted indicator, where DCFH‐DA fluorescence intensity directly correlates with ROS concentration. Following the in vitro PD model protocol, PC‐12 cells were treated with the three distinct NCs for a combined exposure and incubation time (t_exp_ + t_inc_ = 5 h + 24 h). Post‐treatment, cells underwent a double PBS wash and were incubated with 0.5 mL of 0.1% DCFH‐DA (Beyotime, #S0033S, China) in serum‐free DMEM for 30 min at 37 °C. Following another PBS wash, cells were exposed to serum‐free DMEM and the fluorescence of DCFH was quantified using both a microplate reader (Envision@2015, PerkinElmer, USA) and an inverted fluorescence microscope (Eclipse Ti2, Nikon, Japan) at specified excitation and emission wavelengths.

Mitochondrial dysfunction, indicative of cellular health and a precursor to apoptosis, was assessed using the JC‐1 probe. JC‐1's aggregation within the mitochondrial matrix results in red fluorescence, which shifts to green as mitochondrial potential decreases. This shift was a critical early indicator of apoptosis. At t_exp_ + t_inc_ = 5 h + 24 h, the treated PC‐12 cells were incubated with 0.5 mL of 0.5% JC‐1 (Beyotime, #C2003S, China) in complete DMEM for 20 min at 37 °C, followed by washing and exposure to FBS‐free DMEM. The JC‐1 fluorescence was then analyzed using both a microplate reader and CLSM (LSM900, Zeiss, Germany).

To further elucidate the therapeutic impacts, protein expression levels of α‐syn and TH were analyzed post‐treatment (t_exp_ + t_inc_ = 5 h + 24 h,). PC‐12 cells were lysed, centrifuged to remove debris, and the supernatants were assayed for protein concentration using the Bradford method (Beyotime, #P0006C, China). Forty micrograms of proteins were separated via 12% SDS‐PAGE. The gels were subsequently transferred onto polyvinylidene fluoride(PVDF) membranes using standard procedures. The PVDF membranes were then subjected to immunoblotting them with primary and secondary antibodies. Immunoreactive bands were visualized using enhanced chemiluminescence (Beyotime, #P0018AS, China) and captured with a gel documentation system. The following antibody dilutions were used: 1:1000 for rabbit polyclonal antibodies against α‐syn (Abacm, #ab51253, UK), 1:5000 for rabbit polyclonal antibodies against TH (Abcam, #ab137869, UK), 1:2000 for rabbit polyclonal antibodies against β‐actin (Proteintech, #120536‐1‐AP, China), and 1:2000 for goat anti‐rabbit IgG (H+L) peroxidase/HRP‐conjugated secondary antibodies (Elabscience, #E‐AB‐1003, China).

Lastly, the release of DA monomers from NCs was quantified in PC‐12 cells under various conditions, including neutral and acidic media, with and without MPP^+^. PC‐12 cells were cultured in 24‐well plates, each well possessing a surface area of 1.9 cm^2^ and containing 150,000 cells in 1 mL of medium. The day following seeding, the medium was adjusted to a pH of 6.4 by incorporating 1 m HCl into serum‐supplemented medium. Additionally, a specialized medium (pH 6.4 + MPP^+^) was prepared by diluting MPP^+^ reagent into complete DMEM to achieve a final concentration of C_MPP+_ = 3 mm. The cells were then treated with three different NCs at a dosage of C_DA_ = 50 µg mL^−1^, utilizing complete DMEM, pH‐adjusted medium (pH 6.4), and the pH 6.4 medium supplemented with MPP^+^, for a duration of 5 h. The supernatant was collected after treatments, and the cells underwent two PBS washes. Cell detachment was facilitated using 0.05% trypsin/EDTA, applied in a volume of 0.1 mL. Subsequent to trypsin removal, 0.3 mL of medium was added to facilitate the collection of cell pellets through centrifugation at 400 g for 8 min. After a double PBS wash, the cell pellets were resuspended in 0.1 mL of PBS. A handheld homogenizer was employed to disrupt the cell samples, preparing them for analysis. The quantification of DA release was conducted using a DA ELISA Kit (CUSABIO, # CSB‐E08661m, China), adhering strictly to the provided manufacturer's protocol. This approach enabled the precise measurement of DA concentrations in both the supernatant and cell lysates, providing insight into the efficiency of DA monomer release from the NCs under different experimental conditions.

### Gadolinium Labeling through Chelation Reaction

For the Gd labeling of NCs, CRDA, or HRDA was dissolved in 1 mL of citric acid‐Na_2_HPO_4_ buffer (pH 7.4) at a concentration of 22 µM. Subsequently, 230 µM of GdCl_3_·6H_2_O was prepared in 9 mL of the same buffer. The NC solutions were then incrementally added to the GdCl_3_·6H_2_O solution. The mixture was vigorously stirred and allowed to react for 24 h at RT. Following this, unbound Gd^3+^ ions were removed via membrane dialysis using a membrane with a 5 kDa molecular weight cutoff. The Gd‐labeled NCs were subsequently collected through lyophilization. The hydrodynamic size (d_h(N)_) and surface zeta potential (Iζ) of the Gd‐labeled CRDA (Gd‐CRDA) and Gd‐labeled HRDA (Gd‐HRDA) were determined DLS as outlined in previous sections. The quantification of Gd content within the labeled NCs was performed using ICP‐MS. Samples for ICP‐MS analysis were prepared by digesting the NCs in aqua regia, followed by appropriate dilution. The digested and diluted samples were then analyzed by ICP‐MS to determine the concentration of Gd present.

### In Vivo Brain Targeting Ability of NCs

All animal procedures were approved by the Committee on Experimental Animal at Central South University (grant number: CSU‐2023‐0273). To establish a PD model in mice, MPTP (C_MPTP_ = 22.5 mg kg^−1^, Saline with 6% DMSO, Macklin, #M917275, China) was administered via intraperitoneal injection over a period of 13 days. The brain‐targeting capability of the NCs was then assessed. Cy5.5‐labeled NCs were administered intravenously at a dosage of C_DA_ = 600 µg kg^−1^ to male C57BL/6J mice (20–22 g) for durations of 3, 5, and 7 h. The mice were purchased from Hunan SJA Laboratory Animal Co., Ltd. The in vivo fluorescence attributed to Cy5.5 was monitored using an IVIS Lumina II system (Perkin Elmer, USA).

For in vivo brain MRI, Gd‐CRDA, and Gd‐HRDA (C_DA_ = 600 µg kg^−1^) were introduced to the mice via tail vein injection. MRI scans of the mouse brain were performed on a 3 Tesla MR scanner (GE, USA) at post‐injection times of 5, 12, and 24 h. T1‐weighted imaging was conducted using a conventional sequence with the parameters: repetition time (TR) = 679 ms, echo time (TE) = 9.5 ms, matrix size = 256 × 256, field of view (FOV) = 31 × 16 mm^2^, slice thickness = 1 mm, and number of slices = 18. For assessing the concentration‐dependent effects of Gd‐labeled NCs, MRI scans were also carried out under the following conditions: TR = 773 ms, TE = 10 ms, matrix size = 256 × 256, FOV = 29 × 14 mm^2^, slice thickness = 1 mm, and number of slices = 20. The data was analyzed by DOCIM viewer with hotmetal bar. For the NCs, the images were further analyzed by image J to calculate the standard curve.

Subsequently, the Gd content within the brain tissue was quantified using ICP‐MS. Brain tissues were harvested 5 h post‐injection of Gd‐CRDA and Gd‐HRDA, weighed, and digested in 4 mL of nitric acid over 3 days. A 40 µL aliquot of the digested sample was diluted with ultrapure water to a final volume of 2 mL for ICP‐MS analysis.

To identify specific brain regions involved in the uptake of Cy5.5‐labeled CRDA and HRDA, brain tissue was processed post 5‐h exposure. Mice were perfused with PBS followed by 4% paraformaldehyde (PFA), and the brains were excised, fixed in 4% PFA containing an anti‐fading agent (Beyotime, #P0128, China) embedded in OCT compound, and sectioned into 9 µm slices. These slices were then examined using confocal laser scanning microscopy (CLSM) (LSM900, Zeiss, Germany).

### Behavioral Testing

To establish a PD model, MPTP was administered through daily intraperitoneal injections at a dosage of 22.5 mg kg^−1^. Concurrently, the NCs were intravenously administered to the MPTP‐treated mice at a concentration of C_DA_ = 600 µg kg^−1^ every 3 days. For the quantification of nanocapsules, the DA concentration was analyzed by HPLC, which was provided in supporting information. Behavioral assessments were conducted alongside these procedures. For the screening of behavioral data, the data of mice with obvious behavioral abnormalities were removed, and at the same time, the maximum and minimum values were eliminated.

### Morris Water Maze

The Morris water maze test was performed using a circular pool (120 cm in diameter) filled to a depth of 40 cm with water maintained at 18–19 °C. The water was rendered opaque using the food additive titanium dioxide to obscure a submerged platform (10 cm in diameter), located 1 cm below the water surface. The pool was segmented into four quadrants: northeast (NE), northwest (NW), southeast (SE), and southwest (SW), with the platform placed in the SW quadrant.

Over a training period of 4 days, the mice underwent 3 trials daily, released from the NE, NW, and SE positions into the pool. A minimum interval of 30 min was ensured between each trial. Mice that located the platform within 60 s were permitted to remain on it for 5 s. Conversely, mice unable to find the platform within this timeframe were guided to it and allowed to stay for 15 s to ensure familiarity with its location.

On the day following the last training session, the platform was removed for the testing phase. Mice were released from the quadrant farthest from the original platform location into the pool for a probe test, entailing a 60 s free swim in the absence of the platform. Their performance was captured on video and subsequently analyzed using Smart V03 software to evaluate their memory, learning capabilities, and locomotor activity.

### Rotarod Test

Prior to the actual testing, mice underwent a two‐day training regimen consisting of three trials each day. During both training and testing, the mice were placed on a rotarod cylinder that accelerated from 4 to 40 rpm over a 5 min period. On the day of the test, the procedure was replicated, and the time taken for the mice to fall off the cylinder was recorded over three trials, measuring their motor coordination and balance.

### Open Field Test

For the open field test, each mouse was introduced to a black, open‐topped arena measuring 60 cm by 40 cm by 30 cm, situated in a quiet room. The test was preceded by a 3 min habituation period to reduce stress, followed by a 5 min testing session. Movements were tracked and analyzed using Smart V03 software, focusing on the distance, central time, and speed to assess locomotor activity and exploratory behavior. All sessions were recorded for subsequent analysis.

### Pole Test

The pole test involved a vertical pole 75 cm in height and 1 cm in diameter. Mice were placed at the top of the pole facing upwards and the time it took for them to descend to the base was measured. To familiarize the mice with the test, a two‐day training period was implemented, consisting of three trials each day. On the testing day, the descent times were recorded, with a maximum cutoff time of 120 s to conclude the test and finalize recordings. This test aimed to evaluate the mice's motor coordination and agility.

### Histological Assays

The mice underwent perfusion with PBS followed by 4% PFA. Subsequently, their brains were extracted, embedded in both paraffin and OCT compound, and then sectioned into slices of 4 and 9 µm thickness, respectively. Major organs including the heart, liver, spleen, lung, and kidney were excised for further examination.

The prepared tissue sections underwent several staining procedures: IHC staining was performed to visualize specific proteins; ROS were detected using fluorescent staining to assess oxidative stress; and immunofluorescence (IF) staining was employed to visualize within the tissues; HE was used to check the state of major organs. The detailed protocols are provided in Supporting Information.

### Reverse Transcription Quantitative Polymerase Chain Reaction (RT‐PCR)

The striatum and substantia nigra were separated from the whole brain. For the RT‐qPCR assay, total RNA was isolated from the hippocampus using the Trizol™ Reagent (Life Technologies, Carlsbad, USA). The cDNA synthesis was carried out utilizing the RevertAid First Strand cDNA Synthesis Kit (Thermo Fisher Scientific, #K1622, USA). The specific sequences of the primers used for this assay will be detailed subsequently. The RT‐qPCR reactions were performed employing the Master Mix (Novoprotein, #E099‐01A, China). The QuantStudio 6 Flex Real‐Time PCR System (Applied Biosystems, Life Technologies, USA) was utilized to quantify the relative RNA expression levels of the target genes, applying the 2^−ΔΔCt^ method for analysis. Glyceraldehyde 3‐phosphate dehydrogenase (GAPDH) served as the endogenous control, providing a baseline for normalization of the gene expression data. The primer pairs were as follows:

**Table:** 

	Primer sequence(5'to3')
DAT	forward TTTCTCCTGTCCGTCATTGGC
	reverse AGCCCACACCTTTCAGTATGG
TH	forward GAAGGGCCTCTATGCTACCCA
	reverse TGGGCGCTGGATACGAGA
DRD2	forward CAACACCAAGCGTAGCAGC
	reverse TGGTGCTTGACAGCATCTCC
BDNF	forward CAGGGGCATAGACAAAAG
	reverse GAGGGGCCATCCACAGTCTT
SNCA	forward AGAAAACCAAGCAGGGTGTG
	reverse TCCAGGATTCCTTCCTGTGG
ATF6	forward CTTGGCAGCACCTTGACCTTC
	reverse TGGCTCGGCAGGGAGAAATG
GAPDH	forward GCCAAGGTCATCCATGACAACT
	reverse GAGGGGCCATCCACAGTCTT

### In Vivo Western Blot Analysis

The striatum and substantia nigra regions were meticulously dissected from the mouse brain. Protein concentrations within these brain areas were determined using the Bradford assay (Beyotime, #P0006C, China). For electrophoresis, 40 µg of protein from striatum samples and 20 µg from substantia nigra samples were resolved by SDS‐PAGE. The separated proteins were then transferred onto PVDF membranes following standard protocols. The PVDF membranes underwent immunoblotting, where they were incubated with both primary and secondary antibodies. The detection of immunoreactive bands was achieved through enhanced chemiluminescence (Beyotime, #P0018AS, China) and documentation using a gel imaging system. The utilized primary antibodies included: rat monoclonal anti‐DA transporter (1:30, Santa Cruz, #sc‐32258, USA), rabbit polyclonal anti‐TH (1:5000, Abcam, #ab137869, UK), mouse monoclonal anti‐DRD2 (1:30, Santa Cruz, #sc‐32258, USA), rabbit monoclonal anti‐BDNF (1:1000, Abcam, #ab108319, UK), rabbit polyclonal anti‐α‐syn (1:1000, Abcam, #ab51253, UK), rabbit polyclonal anti‐ATF6 (1:1000, Proteintech, #24169‐1‐AP, China), rabbit polyclonal anti‐β‐actin (1:2000, Proteintech, #120536‐1‐AP, China), and rabbit polyclonal anti‐GAPDH (1:2000, Affinity, #AF7021, China).Secondary antibodies were applied as follows: goat anti‐rabbit IgG (H+L) peroxidase/HRP‐conjugated (1:2000, Elabscience, #E‐AB‐1003, China), goat anti‐mouse IgG (H+L) peroxidase/HRP‐conjugated (1:2000, Elabscience, #E‐AB‐1001, China), and goat anti‐rat IgG (H+L) peroxidase/HRP‐conjugated (1:2000, Elabscience, #E‐AB‐1041, China), facilitating the visualization of specific protein targets related to dopamine signaling and neuroprotection within the brain.

## Conflict of Interest

The authors declare no conflict of interest.

## Supporting information



Supporting Information

Supplemental Movie 1

Supplemental Movie 2

Supplemental Movie 3

Supplemental Movie 4

Supplemental Movie 5

Supplemental Movie 6

Supplemental Movie 7

Supplemental Movie 8

Supplemental Movie 9

Supplemental Movie 10

Supplemental Movie 11

Supplemental Movie 12

Supplemental Movie 13

Supplemental Movie 14

Supplemental Movie 15

Supplemental Movie 16

Supplemental Movie 17

Supplemental Movie 18

Supplemental Movie 19

Supplemental Movie 20

Supplemental Movie 21

Supplemental Movie 22

Supplemental Movie 23

Supplemental Movie 24

Supplemental Movie 25

Supplemental Movie 26

Supplemental Movie 27

Supplemental Movie 28

Supplemental Movie 29

Supplemental Movie 30

Supplemental Movie 31

Supplemental Movie 32

Supplemental Movie 33

Supplemental Movie 34

Supplemental Movie 35

Supplemental Movie 36

Supplemental Movie 37

Supplemental Movie 38

Supplemental Movie 39

Supplemental Movie 40

Supplemental Movie 41

## Data Availability

The data that support the findings of this study are available in the supplementary material of this article.
