# Supplementary material for: Parkinson Disease ‐Targeted Nanocapsules for Synergistic Treatment: Combining Dopamine Replacement and Neuroinflammation Mitigation
Source: Adv Sci (Weinh). 2024 Oct 21;11(46):2404717. doi: 10.1002/advs.202404717 (PMC11633476; doi:10.1002/advs.202404717)
Supplement: Supplementary file 1 — Supporting Information [file ADVS-11-2404717-s008.pdf]

ADVANCED SCIENCE
Open Access

Supporting Information

for *Adv. Sci.*, DOI 10.1002/advs.202404717

Parkinson Disease -Targeted Nanocapsules for Synergistic Treatment: Combining Dopamine Replacement and Neuroinflammation Mitigation

Ziyao Liu, Shijun Xiang, Bei Chen, Jian Li, Dingcheng Zhu, Hongjuan Xu and Shuo Hu**

Parkinson Disease-Targeted Nanocapsules for Synergistic Treatment: Combining Dopamine Replacement and Neuroinflammation Mitigation

Ziyao Liu[a,c], *Shijun Xiang*[a], *Bei Chen*[a], *Jian Li*[a], *Dingcheng Zhu*[d], *Hongjuan Xu*[a,b,c]*

Shuo Hu[a,b,c]*

[a]Department of Nuclear Medicine, Xiangya Hospital, Central South University, Changsha 410008, China

[b]National Clinical Research Center for Geriatric Diseases, Xiangya Hospital, Central South University, Changsha 410008, China

[c]Key Laboratory of Biological Nanotechnology of National Health Commission, Xiangya Hospital, Central South University, Changsha 410008, China

[d]College of Material, Chemistry and Chemical Engineering, Key Laboratory of Organosilicon Chemistry and Material Technology, Ministry of Education, Hangzhou Normal University Hangzhou 311121, China

*Email: angelaxhjcsu@csu.edu.cn，Hushuoxy@csu.edu.cn

Quantification of Components within Nanocapsules (NCs)

For the quantification of components within the NCs, various methods were employed. Dopamine (DA) levels were determined after immersing the NCs in 1 M HCl for 3 days. The DA was then isolated using an ultracentrifuge filter (cutoff molecular weight of 5 kDa) at 8000 rpm for 30 min. The eluents were collected and quantified via High-Performance Liquid Chromatography (HPLC) with a two-solvent gradient: Solvent A (0.02 M KH_2PO_4 and 1 M HCl at pH 3.5) and Solvent B (100% methanol). These solvents were pre-filtered through a 0.22 μm membrane. The HPLC run began with 95% Solvent A and 5% Solvent B for the first 10 min, followed by a shift to 5% Solvent A and 95% Solvent B from 10 to 15 min. The flow rate was maintained at 1 mL/min, with signals detected at a wavelength of 280 nm. A calibration curve for DA, ranging from 63.5 to 1.95 μg/mL, was prepared with DA monomers (Aladdin, #S111161, China) in ultrapure water, and DA content was derived from the area under the curve at 2-2.5 min relative to the standard.

Protein concentration within the NCs was quantified using a Bradford assay (Beyotime, #P0006C, China). The concentration of boron was measured by digesting the NCs with an aqua regia solution, followed by inductively coupled plasma mass spectrometry (ICP-MS) analysis. To quantify the cRGD levels, the NCs were reacted with phenanthrenequinone. A mix of 50 μL of sample, 150 μL of phenanthrenequinone (Aladdin, #S P106382, China ,150 μM in ethanol), and 25 μL of 2 M NaOH was incubated at 60 °C for 3 h. Subsequently, 200 μL of the mixture was neutralized with 200 μL of 1 M HCl and kept in the dark for 1 h at room temperature (RT). Fluorescence was measured using a plate reader (Ex: 312 nm, Em: 395 nm). A standard curve was created using cRGD, CAT, and HSA at concentrations ranging from 250 to 3.9 μg/mL.

The total fluorescence intensity of each sample (I_{total}) was measured, and the protein content was deduced using a Bradford assay (Beyotime, #P0006C, China). The protein-related fluorescence intensity ($I_{protein}$) was calculated using the calibration curve for catalase (CAT) or human serum albumin (HSA). The fluorescence intensity attributable to cRGD (I_{RGD}) was obtained by subtracting $I_{protein}$ from I_{total}. The concentration of cRGD was then calculated using the standard cRGD curve.

Fluorescence Labeling of Cy5.5

For the fluorescence labeling process, Cy5.5 NHS ester (Aladdin, #C171354, China) was prepared by dissolving it in dimethylformamide (DMF) to achieve a concentration of 1 mg/mL. Concurrently, solutions of CAT-cRGD and CAT alone were prepared by dissolving in Tris-HCl buffer (pH = 9.5) to a final concentration of 10 mg/mL (equivalent to 50 mg in 5 mL, translating to 41.6 μM). It is important to note that although the conjugation of cRGD to CAT results in an increase in the molecular weight of CAT, the calculations for fluorescence labeling were based on the original molecular weight of CAT.

The Cy5.5 NHS solution, amounting to 447 μL (124.8 μM of Cy5.5 NHS), was gradually added to the CAT or CAT-cRGD solutions under mild stirring conditions. This mixture was continuously stirred for a duration of 5 h to facilitate the labeling reaction. To remove unbound fluorophores, the mixture was subjected to membrane dialysis using a membrane with a 100 kDa molecular weight cutoff. Following dialysis, lyophilization was employed to derive the final products, CAT-Cy5.5 and CAT-cRGD-Cy5.5, respectively. The same procedure was replicated to produce HSA-Cy5.5. Given that the concentration of HSA in the NCs is higher than that of CAT, the HSA to Cy5.5 molar ratio was adjusted to 1:1 for optimal labeling efficiency. The fluorescence intensity of Cy5.5 within the NCs was quantitatively measured using a fluorescence meter (HORIBA Fluoromax-4, Horiba Scientific, Japan), specifically at an excitation wavelength of 678 nm.

Cytotoxicity Assays

For the assays, PC-12 and bEnd.3 cells were plated at a density of 30,000 cells/well in 96-well plates, each well having a growth area of 0.32 cm^2 and containing 150 μL of the medium. Following overnight incubation, these cells were subjected to various treatments for 24 and 48 h. Post-treatment, a single wash with 150 μL of PBS was performed before adding 100 μL of a 10% resazurin solution (0.25 mg/mL, Aladdin, #R105538, China) in complete cell medium for a further 4-hour incubation. The optical density (OD) of each well was measured at 570 nm and 600 nm using a microplate reader (Epoch BioTek, USA), where the OD values are directly correlated with the number of viable cells. The OD readings obtained from cells treated with the samples were normalized against a control medium containing resazurin but not exposed to cells or samples. Cell viability was then calculated using the following equation:

$$\%\mathbf{Reduction\ of\ resazurin\ agent} = \frac{(\mathbf{E_{oxi600}} \times \mathbf{A_{570}}) - (\mathbf{E_{oxi570}} \times \mathbf{A_{600}})}{(\mathbf{E_{red570}} \times \mathbf{C_{600}}) - (\mathbf{E_{red600}} \times \mathbf{C_{570}})}$$

Where E_{oxi570} and E_{oxi600} are the molar extinction coefficients of oxidized resazurin agent at 570 nm and 600 nm, respectively (E_{oxi570} = 80,586, E_{oxi600} = 117,216); E_{red570} and E_{red600} are the molar extinction coefficients of reduced resazurin agent at 570 nm and 600 nm, respectively (E_{red570} = 155,677, E_{red600} = 14,652); A_{570} and A_{600} represent the absorbance of the samples at 570 nm and 600 nm; C_{570} and C_{600} represent the absorbance of negative controls (cell medium with resazurin agent) at 570 nm and 600 nm.

To simulate Parkinson disease (PD) conditions, PC-12 cells were exposed to MPP^+ at various concentrations (C_{MPP^+} = 1,3,5,7,9 mM). NCs at a concentration of C_{DA} = 50 μg/mL were then applied to these PD-model PC-12 cells for 24 h. Cell viability in response to these treatments was measured using the same resazurin assay method described above.

Time-Dependent Cellular Uptake Characterized by Flow Cytometry

In our investigation, we utilized flow cytometry (BD LSR Fortessa, Biosciences, USA) to quantify the cellular uptake of Cy5.5-labeled NCs: CRDA-Cy5.5, CDA-Cy5.5, and HRDA-Cy5.5. bEnd.3 cells were cultured at a density of 150,000 cells/mL in 1 mL of medium within 24-well plates (each with a growth area of 1.9 cm^2), and left to adhere overnight. The cells were then exposed to these NCs at a concentration of C_{DA} = 50 μg/mL, assessing the time-dependent endocytosis over periods of 1, 3, 5, and 7 h. The cellular uptake was monitored by measuring the fluorescence intensity of cells at these intervals using flow cytometry. After each time point, the medium was discarded, and the cells were washed with 1 mL of PBS twice. Detachment was achieved by treating the cells with 0.1 mL of 0.05% trypsin/EDTA solution (Procell, China), followed by neutralization with 0.4 mL of complete medium. The cell suspension was centrifuged at 400 g for 8 min, the pellet was resuspended in 0.2 mL of PBS, and subsequently analyzed using flow cytometry. For analysis, 10,000 gated cells per sample were recorded

mRNA Sequencing Analysis

Total RNA was extracted from the hippocampal region of mouse brains utilizing Trizol™ Reagent (Life Technologies, Carlsbad, USA). Subsequent to this, mRNA was isolated from the total RNA sample employing poly-T oligo-attached magnetic beads. The synthesis of first-strand cDNA was initiated with a random hexamer primer and M-MuLV Reverse Transcriptase, followed by the generation of second-strand cDNA using DNA Polymerase I and a mix of dNTPs. To select for cDNA fragments in the range of 370–420 bp, the AMPure XP system (Beckman Coulter, Beverly, USA) was deployed, which were then amplified via PCR. The PCR products were further purified using AMPure XP beads to construct the sequencing library.

The integrity and quality of the prepared library were assessed using the Qubit 2.0 Fluorometer and an Agilent 2100 bioanalyzer. Upon verification of the expected insert size, the library concentration was quantified through RT-qPCR. Libraries were then pooled based on their effective concentration and the desired sequencing output before being subjected to sequencing on the Illumina NovaSeq 6000 platform.

Differential expression analysis was conducted with the DESeq2 program, applying a scaling factor for normalization of read counts. Significantly differentially expressed genes were identified with a threshold of $p \leq 0.05$ and an absolute log2(fold change) ≥ 1.5. The Kyoto Encyclopedia of Genes and Genomes (KEGG) database facilitated the identification of enriched pathways, using the clusterProfiler R package (version 3.8.1) for analysis. Additionally, Gene Set Enrichment Analysis (GSEA) was utilized as a computational method to discern significant differences between two biological samples, ranking genes to identify enrichment at either end of the gene set list.

Immunohistochemistry (IHC) Staining

The process for immunohistochemistry (IHC) staining commenced with the dewaxing of brain slides. Initially, the slides were incubated at 60°C for 2 h, followed by a series of deparaffinization steps involving xylene treatments (20 min for three cycles) and graded alcohol solutions (two cycles of 5 min in 100% ethanol, then 5 min each in 90%, 80%, and 70% ethanol), concluding with rehydration in ultra-pure water for 5 min. Subsequent to rehydration, the slides were washed thrice with PBS for 5 min each time.

Antigen retrieval was performed by microwaving the slides in EDTA solution (pH adjusted to 9 or 6, as per the antibody manufacturer's instructions) for 20 min, followed by triple rinsing in ultra-pure water for 5 min. The slides were then treated with a 3% hydrogen peroxide solution for 10 min to quench endogenous peroxidase activity.

After washing twice with ultra-pure water and once with PBS for 5 min each, slides were blocked with 10% goat serum (Solarbio, #SL038, China) for 1 h at RT. Primary antibodies were then applied at the following dilutions: 1:200 for rabbit polyclonal antibodies against α-synuclein (Abcam, #ab51253, UK), 1:200 for rabbit polyclonal antibodies against tyrosine hydroxylase (TH) (Abcam, #ab137869, UK), 1:500 for rabbit polyclonal antibodies against GFAP (Abacm, #ab7260, UK),and 1:500 for rabbit polyclonal antibodies against Iba1 (Proteintech, #10904-1-AP, China), and incubated overnight at 4°C. The subsequent day, slides were warmed to 37°C for 30 min to allow the slides to reach RT, then rinsed to remove primary antibodies. Secondary antibodies (Goat Anti-Rabbit IgG (H+L), peroxidase/HRP-conjugated, Elabscience, China) were applied at a 1:400 dilution and incubated for 30 min at 37°C.

Following three washes with PBS for 5 min each, slides were developed using a DAB kit (ZSGB-BIO, China) and rinsed under running water for 3 min. Slides were stained with hematoxylin for 10 min, a step crucial for highlighting the nuclei, followed by a rinse under running water for 3 min to remove excess stain. The differentiation process, involving 1% hydrochloric acid in ethanol for 30 s, helped clarify the hematoxylin staining by removing it from non-nuclear parts. The slides were treated with blueing solution (Servicebio, #G1040, China) for 30 s to enhance the blue hue of the nuclei, followed by another rinse under running water for 2 min. The completion of staining was followed by a systematic dehydration process. This involved immersing the slides in a series of ethanol solutions of descending concentration—twice in 70% ethanol for 5 min, then in 80%, 90%, and 100% ethanol for 5 min each—to progressively remove water from the tissue. Afterward, the slides were cleared in fresh xylene for 10 min to ensure transparency in preparation for mounting. The final step involved sealing the tissue slides with neutral gum to preserve the specimens. For visualization of the brain slides, the Vectra Polaris system (Akoya Bioscience, USA) was employed.

Reactive Oxygen Species (ROS) Fluorescent Staining

ROS fluorescent staining was conducted on frozen brain tissue sections. Initially, the slides were acclimatized to RT for 30 min. This step was followed by treatment with anti-fluorescence quenching agents for 5 min to preserve the fluorescent signal. Subsequently, the slides were rinsed thoroughly under running water for 10 min to remove any residual quenching agent. The primary staining involved incubating the slides with a ROS-specific fluorescence solution (Sigma, #D7008, United States) at 37°C for 30 min. This step allowed for the specific labeling of reactive oxygen species within the tissue. Following this, the slides were counterstained with DAPI solution (Servicebio, #G1012, China) for 10 min at RT, which facilitated the visualization of cell nuclei. After staining, the slides were washed three times with phosphate-buffered saline (PBS) for 5 min each to remove excess stain and to clear the background. Finally, the stained slides were examined using the Vectra Polaris imaging system (Akoya Bioscience, USA), enabling detailed visualization and analysis of ROS presence and distribution within the brain tissues.

Hematoxylin and Eosin (H&E) Staining

The process for H&E staining begins with the dewaxing of tissue slides, following the protocol outlined in the immunohistochemistry (IHC) staining section. Subsequently, the slides are rinsed three times with PBS for 5 min each to prepare for staining. The staining procedure involves applying hematoxylin to the slides for 10 min to stain cell nuclei, followed by rinsing under running water for 3 min to remove excess stain. The slides are then differentiated in 1% hydrochloric acid ethanol for 30 s to refine the hematoxylin staining and treated with blueing solution (Servicebio, #G1040, China) for 30 s to enhance the nuclear contrast. After another rinse under running water for 2 min, the slides are stained with eosin (Biosharp, #BL700A-2, China) for 2 min, providing cytoplasmic and extracellular matrix contrast by coloring these components pink, followed by rinsing under running water for 3 min to remove excess stain. Following staining, the slides undergo dehydration using the methodology described in the IHC staining section, progressing through graded concentrations of ethanol and xylene. The final step involves sealing the tissue slides with neutral gum to preserve the stained tissue. Imaging of the stained major tissue slides is performed using a microscope (Zeiss, Germany), enabling the detailed examination of cellular structures and morphology essential for histopathological analysis

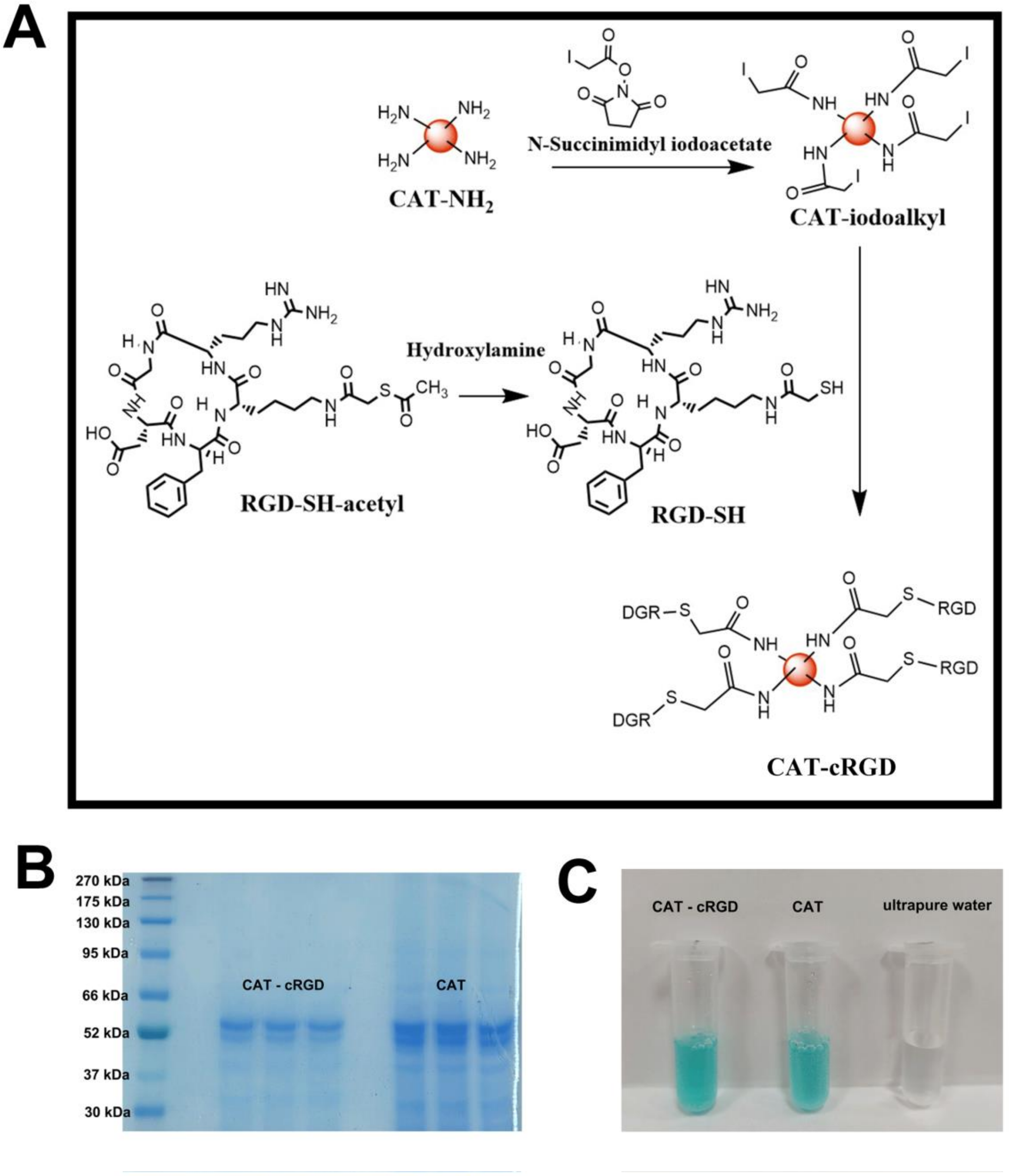

Figure S1. Characterization of cRGD-Modified CAT (CAT-cRGD) and Comparative Analyses. (A) Schematic of the synthetic procedure for CAT-cRGD. (B) SDS-PAGE analysis comparing 40 μg of CAT-cRGD and CAT. (C) Reaction mixture containing 3,3′,5,5′-tetramethylbenzidine (C_{TMB} = 2.5 mM), hydrogen peroxide (C_{H2O2} = 88.2 mM), and either CAT-cRGD or CAT alone (both at 1 mg/mL) in NaOAc-HAc buffer at pH 5.

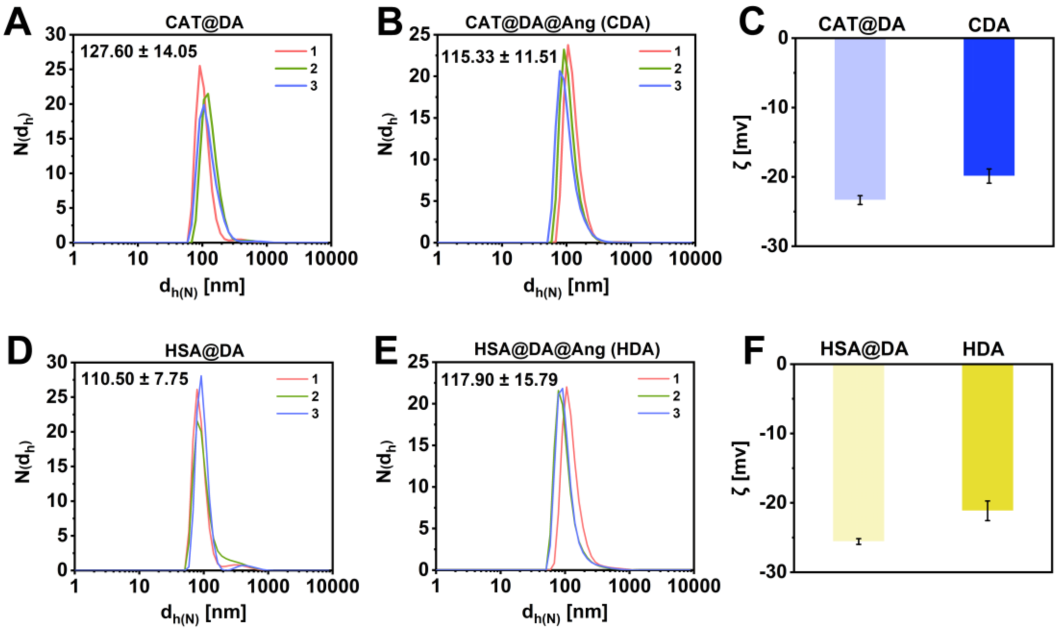

Figure S2. Hydrodynamic Diameter and ζ-Potential Measurements. Hydrodynamic diameter distribution N(d_h) for (A) CAT@DA, (B) CDA, (D) HSA@DA, (E) HDA, and ζ-potential for (C) CAT@DA and CDA, (F) HSA@DA and HDA.

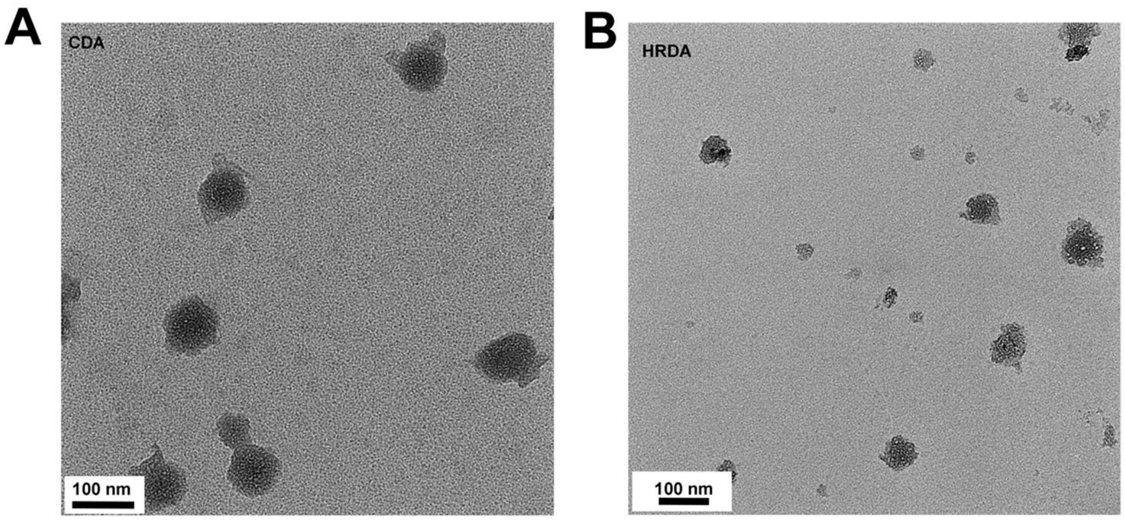

Figure S3. TEM images of CDA and HRDA at C_{NCs} = 1 mg/mL.

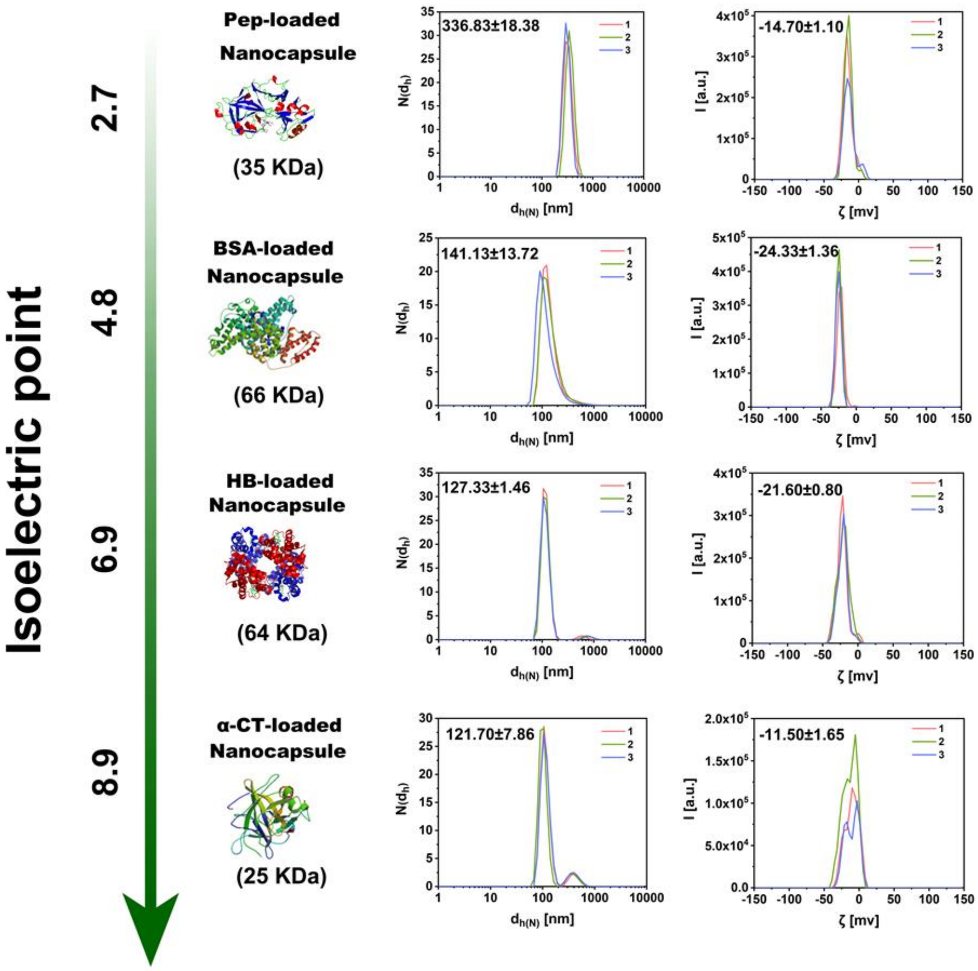

Figure S4. Compilation of data from Figure 1E. The number distribution of hydrodynamic diameters N(d_h) and the distribution of ζ-potential I(ζ) of NCs loaded with (A) Pep, (B) BSA, (C)HB and (D) α-CT at a concentration C_{NCs} = 1 mg/mL.

Table S1. The loading efficiencies of different proteins in NCs.

	Pep-loaded nanocapusle	BSA-loaded nanocapusle	HB-loaded nanocapusle	α-CT-loaded nanocapusle
$C_{protein}$ /C_{NCs}	0.47%	4.55%	17.17%	22.82%

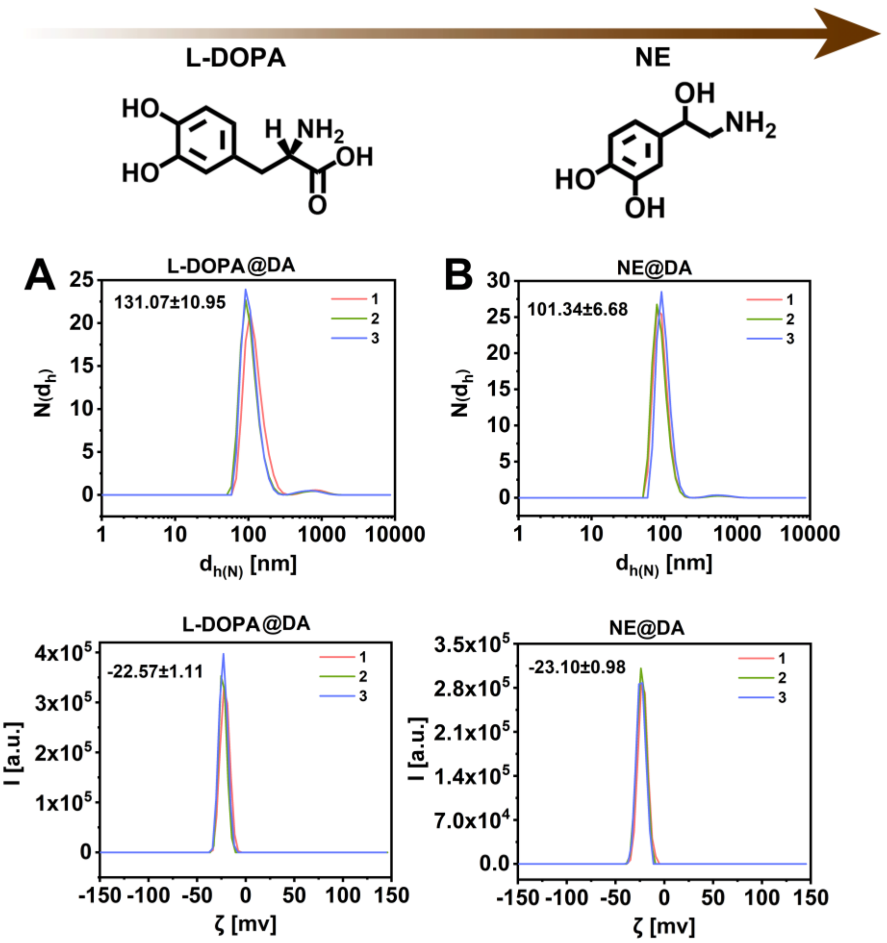

Figure S5. Compilation of data from Figure 1E. The number distribution of hydrodynamic diameters $N(d_h)$ and the distribution of ζ-potential I(ζ) of NCs constructed by (A) L-DOPA and (B) NE at a concentration C_{NCs} = 1mg/mL.

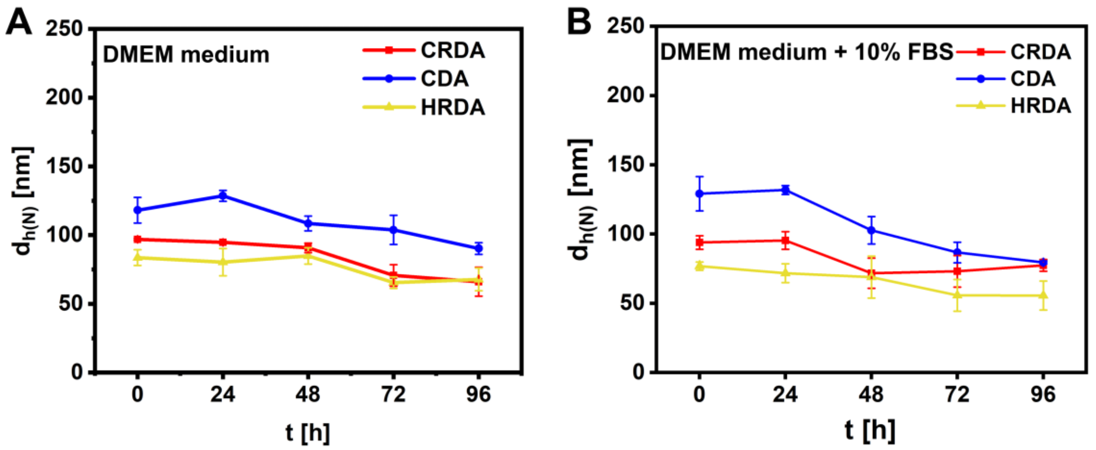

Figure S6. Stability of NCs in DMEM and in DMEM supplemented with 10% fetal bovine serum, assessed by hydrodynamic size $d_{h(N)}$ at a concentration of C_{NCs} = 1 mg/mL.

The results demonstrate that the nanoparticles keep their stability for up to 48 h in cell medium and 24 h in FBS-supplemented cell medium. Beyond these time points, a slight reduction in the hydrodynamic size of the nanoparticles was observed in both media.

Furthermore, as presented in Figure 4, the nanoparticles exhibited no noticeable MRI signals at 24 h. Considering the stability of the nanoparticles in FBS-supplemented cell medium for 24 h, we believe that this duration is sufficient for the intended therapy.

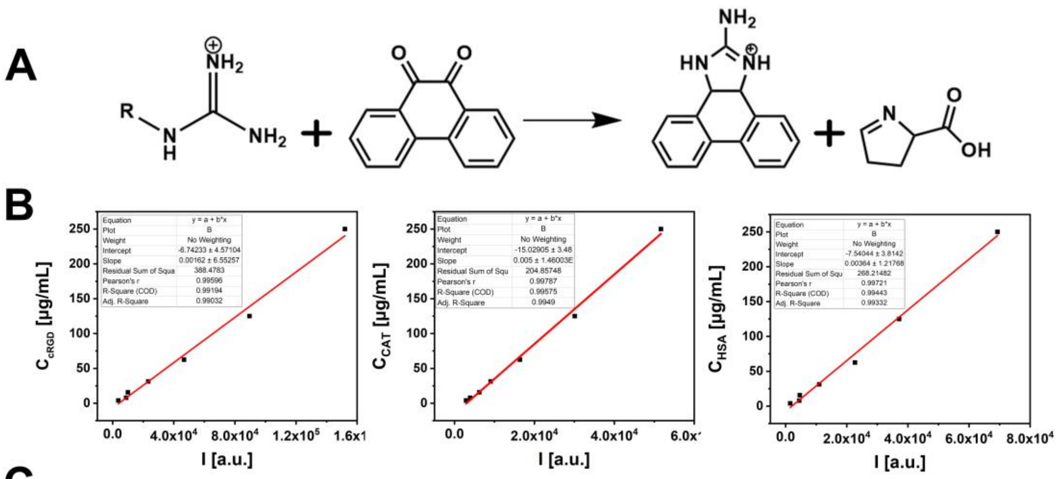

C

	CRDA-1	CRDA-2	CRDA-3		HRDA-1	HRDA-2	HRDA-3
I [a.u.]	36305.70	34321.70	38446.70	I [a.u.]	67834.00	60327.00	57065.00
C_{CAT} [μg/mL]	17.10	14.33	11.31	C_{HSA}[μg/mL]	55.80	47.96	48.70
I_{CAT} [a.u.]	6426.00	5872.48	5268.42	I_{HSA} [a.u.]	17400.73	15248.42	15449.46
I-I_{CAT} [a.u.]	29879.70	28449.22	33178.28	I-I_{HSA} [a.u.]	50433.27	45078.58	41615.54
C_{cRGD}[μg/mL]	41.66	39.35	47.01	C_{RGD} [μg/mL]	74.96	66.28	60.67

Figure S7. Guanidine Group Reaction and Standard Curves. (A) Reaction between guanidine group and phenanthrenequinone. (B) Standard curves of cRGD, CAT, and HSA ranging from 250 to 3.9 μg/mL. (C) Procedure for calculating the cRGD group content within NCs.

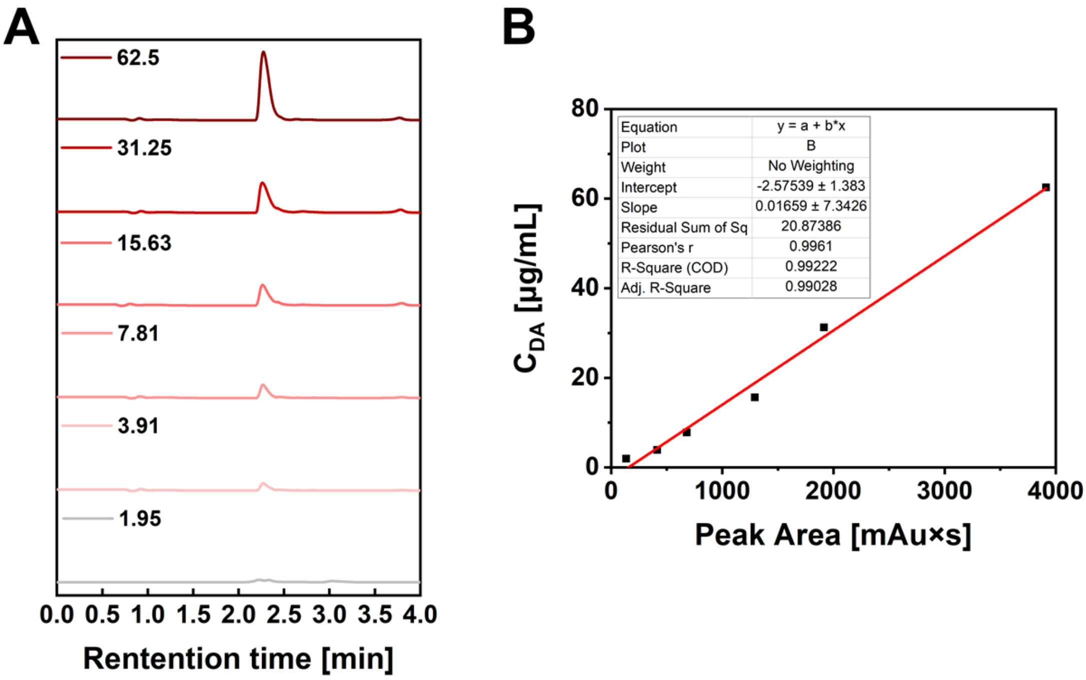

Figure S8. Standard curve of DA for HPLC analysis. (A) The HPLC curve of DA from 62.5 to 1.95 μg/mL. (B) Linear fitting of the peak area of DA determined by HPLC versus the DA. The results are C_{DA} [μg/mL] = 0.017×peak area-2.58, R^2 = 0.9903.

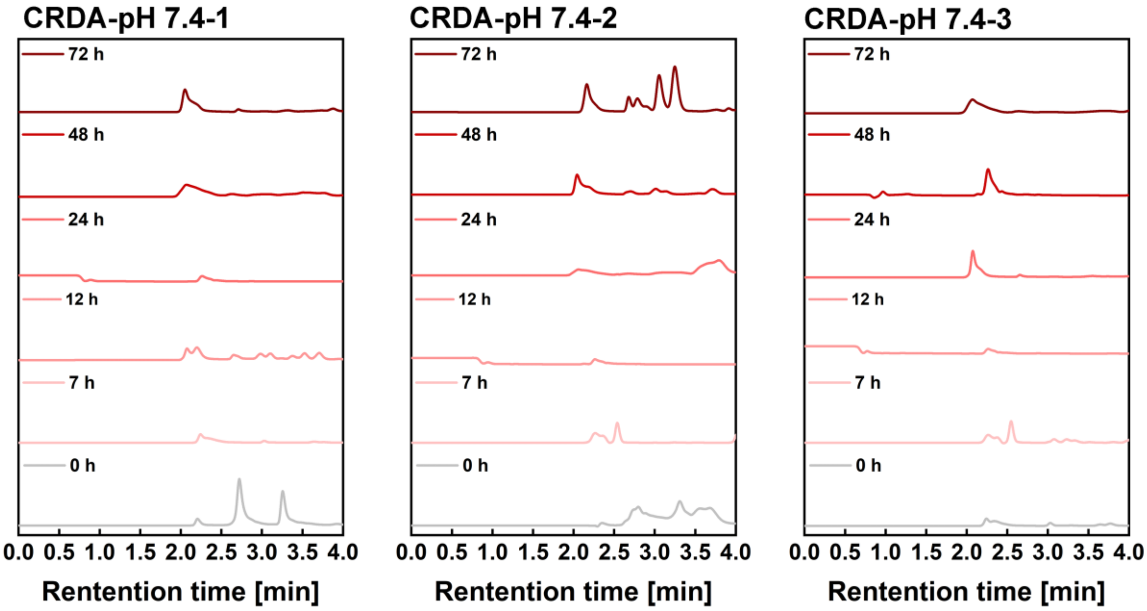

Figure S9. Compilation of data from Figure 1J. CRDA at a concentration of C_{DA} = 50 μg/mL were incubated with pH 7.4 buffer for specific time points. The samples were centrifuged at 8000 rpm for 30 min with an ultracentrifuge filter (cutoff Mw = 3 kDa). The eluents were collected and quantified by HPLC. The results were represented by three independent samples, and the amounts of released DA were quantified through peak area at t = 2-2.5 min according to the standard curve shown in Figure S8.

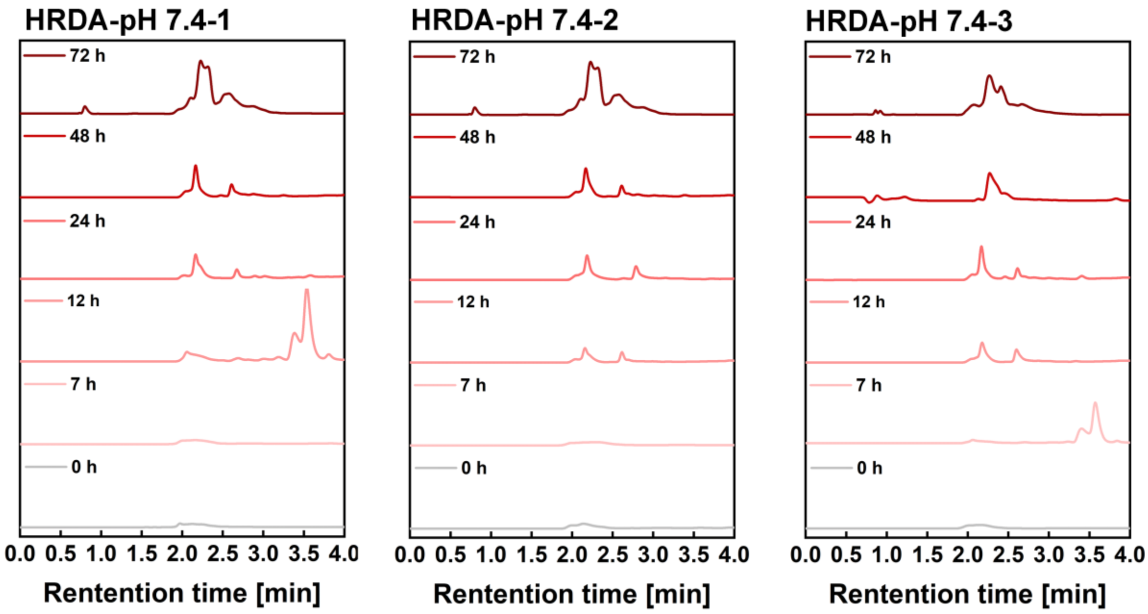

Figure S10. Compilation of data from Figure 1J. HRDA at a concentration of C_{DA} = 50 μg/mL were incubated with pH 7.4 buffer for specific time points. The samples were centrifuged at 8000 rpm for 30 min with an ultracentrifuge filter (cutoff Mw = 3 kDa). The eluents were collected and quantified by HPLC. The results were represented by three independent samples, and the amounts of released DA were quantified through peak area at t = 2-2.5 min according to the standard curve shown in Figure S8.

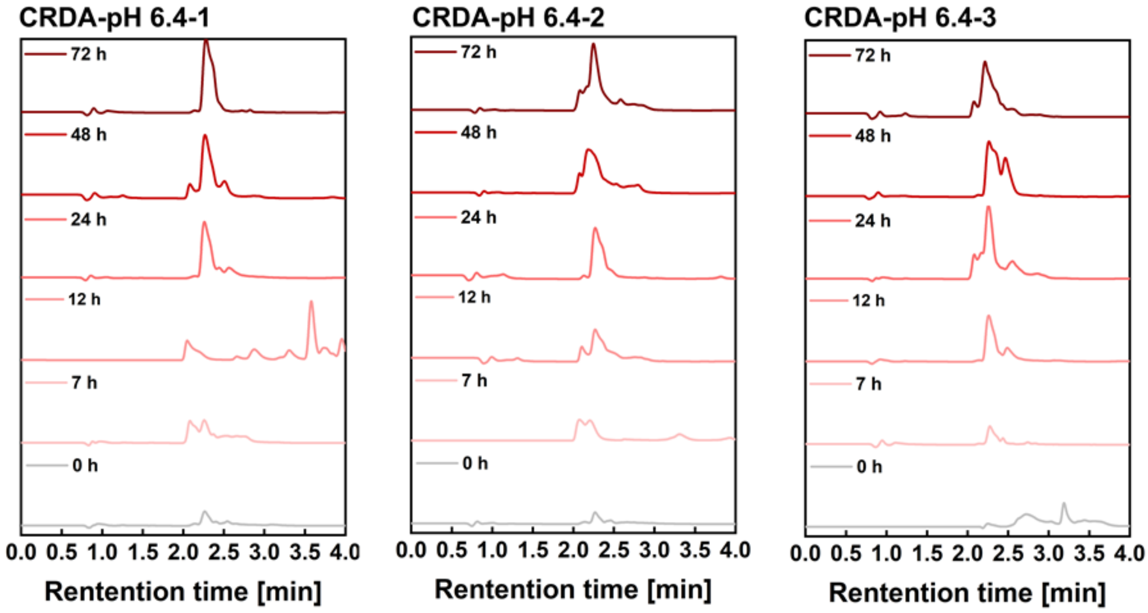

Figure S11. Compilation of data from Figure 1J. CRDA at a concentration of C_{DA} = 50 μg/mL were incubated with pH 6.4 buffer for specific time points. The samples were centrifuged at 8000 rpm for 30 min with an ultracentrifuge filter (cutoff Mw = 3 kDa). The eluents were collected and quantified by HPLC. The results were represented by three independent samples, and the amounts of released DA were quantified through peak area at t = 2-2.5 min according to the standard curve shown in Figure S8.

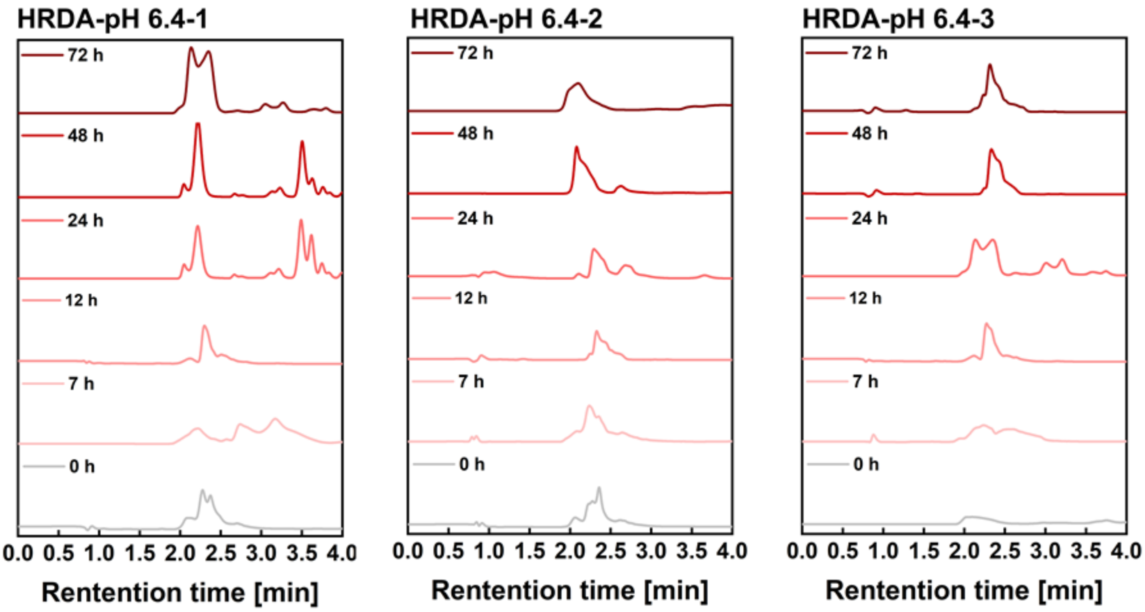

Figure S12. Compilation of data from Figure 1J. HRDA at a concentration of C_{DA} = 50 µg/mL were incubated with pH 6.4 buffer for specific time points. The samples were centrifuged at 8000 rpm for 30 min with an ultracentrifuge filter (cutoff Mw = 3 kDa). The eluents were collected and quantified by HPLC. The results were represented by three independent samples, and the amounts of released DA were quantified through peak area at t = 2-2.5 min according to the standard curve shown in Figure S8.

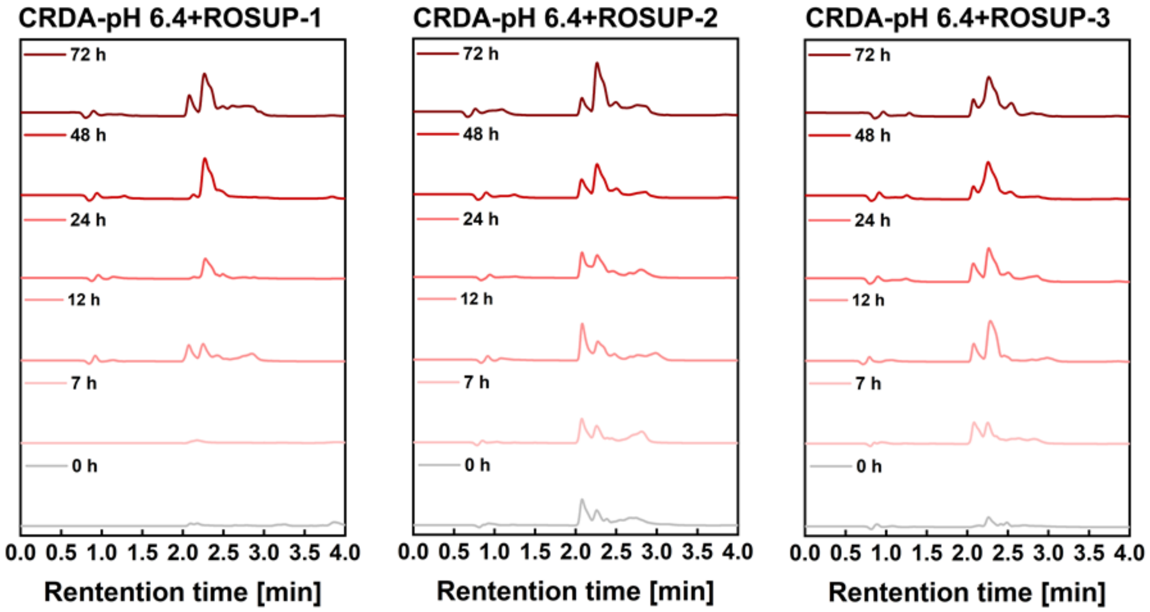

Figure S13. Compilation of data from Figure 1J. CRDA at a concentration of C_{DA} = 50 µg/mL were incubated with pH 6.4 + ROSUP buffer for specific time points. The samples were centrifuged at 8000 rpm for 30 min with an ultracentrifuge filter (cutoff Mw = 3 kDa). The eluents were collected and quantified by HPLC. The results were represented by three independent samples, and the amounts of released DA were quantified through peak area at t = 2-2.5 min according to the standard curve shown in Figure S8.

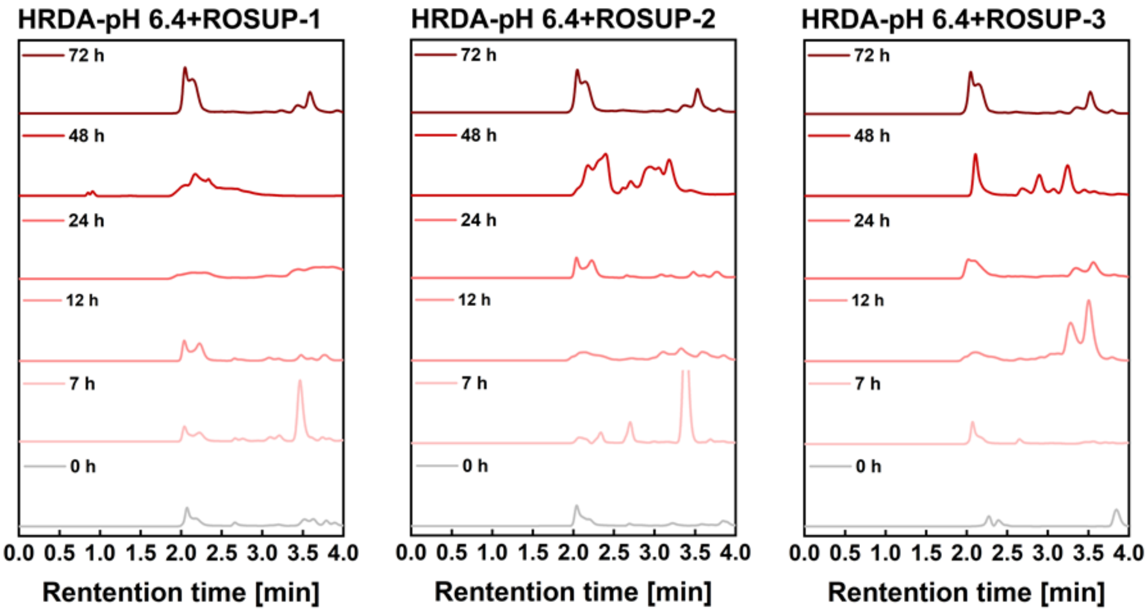

Figure S14. Compilation of data from Figure 1J. HRDA at a concentration of C_{DA} = 50 μg/mL were incubated with pH 6.4 + ROSUP buffer for specific time points. The samples were centrifuged at 8000 rpm for 30 min with an ultracentrifuge filter (cutoff Mw = 3 kDa). The eluents were collected and quantified by HPLC. The results were represented by three independent samples, and the amounts of released DA were quantified through peak area at t = 2-2.5 min according to the standard curve shown in Figure S8.

Table S2. The peak areas and concentrations of released DA over time from NCs at C_{DA} = 50 µg/mL

		**Peak Area [mAu×s]**			**C_{DA} [µg/mL]**				
**CRDA-pH 7.4**	**t [h]**	**1#**	**2#**	**3#**	**1#**	**2#**	**3#**	**Average**	**STEDV**
	72	432.9	425.4	464.9	4.61	4.49	5.14	4.75	0.35
	48	457.4	399	377.6	5.02	4.05	3.69	4.25	0.69
	24	339	354.2	371.3	3.05	3.3	3.59	3.31	0.27
	12	203.5	282.8	231.2	0.8	2.12	1.26	1.39	0.67
	7	194.3	142.3	121.8	0.65	-0.21	-0.55	-0.04	0.62
	0	68.5	39.1	70.8	-1.44	-1.93	-1.4	-1.59	0.29
**HRDA-pH 7.4**	**t [h]**	**1#**	**2#**	**3#**	**1#**	**2#**	**3#**	**Average**	**STEDV**
	72	497.7	497.7	495	5.69	5.69	5.64	5.67	0.03
	48	440.1	431.5	469	4.73	4.59	5.21	4.84	0.33
	24	349.5	410.5	415.7	3.23	4.24	4.33	3.93	0.61
	12	298.8	215.4	322.8	2.38	1	2.78	2.06	0.94
	7	238	254.8	280.8	1.38	1.65	2.09	1.71	0.36
	0	138.6	209	173.2	-0.27	0.89	0.3	0.31	0.58
**CRDA-pH 6.4**	**t [h]**	**1#**	**2#**	**3#**	**1#**	**2#**	**3#**	**Average**	**STEDV**
	72	1463	1243.8	1125.7	21.71	18.07	16.11	18.63	2.84
	48	1209.9	1141.5	1360.6	17.51	16.37	20.01	17.96	1.86
	24	928.6	1357.2	997.3	12.84	19.95	13.98	15.59	3.82
	12	419.7	878.2	749.3	4.39	12	9.86	8.75	3.93
	7	267.9	390.5	248.4	1.87	3.91	1.55	2.44	1.28
	0	170.6	144.4	62.76	0.26	-0.18	-1.53	-0.49	0.93
**HRDA-pH 6.4**	**t [h]**	**1#**	**2#**	**3#**	**1#**	**2#**	**3#**	**Average**	**STEDV**
	72	1217.1	1146.8	1372.1	17.63	16.46	20.2	18.1	1.91
	48	1311.9	1169.4	1193.7	19.2	16.84	17.24	17.76	1.27
	24	829.7	724.8	720.3	11.2	9.46	9.38	10.01	1.03
	12	600.1	770.5	735.8	7.39	10.21	9.64	9.08	1.49
	7	503.8	439.4	640.5	5.79	4.72	8.06	6.19	1.7
	0	342.1	279.2	380.2	3.1	2.06	3.74	2.97	0.85
**CRDA-pH 6.4+ROSUP**	**t [h]**	**1#**	**2#**	**3#**	**1#**	**2#**	**3#**	**Average**	**STEDV**
	72	783.6	800.8	909.9	10.43	10.72	12.53	11.23	1.14
	48	703.5	771.4	857.3	9.1	10.23	11.66	10.33	1.28
	24	341.7	296.3	569.7	3.1	2.34	6.88	4.11	2.43
	12	218.1	328.8	696.7	1.05	2.88	8.99	4.31	4.16
	7	71.2	209.6	238	-1.39	0.9	1.38	0.3	1.48
	0	44.5	156.2	118.4	-1.84	0.02	-0.61	-0.81	0.94
**HRDA-pH 6.4+ROSUP**	**t [h]**	**1#**	**2#**	**3#**	**1#**	**2#**	**3#**	**Average**	**STEDV**
	72	559.5	553.1	522.5	6.71	6.61	6.1	6.47	0.33
	48	518.1	521.7	546.2	6.03	6.08	6.49	6.2	0.25
	24	342	340.8	424.8	3.1	3.08	4.48	3.55	0.8
	12	340.8	359.6	352.7	3.08	3.39	3.28	3.25	0.16
	7	280.5	112.5	306.7	2.08	-0.71	2.52	1.3	1.75
	0	98.6	82.7	98.7	-0.94	-1.2	-0.94	-1.03	0.15

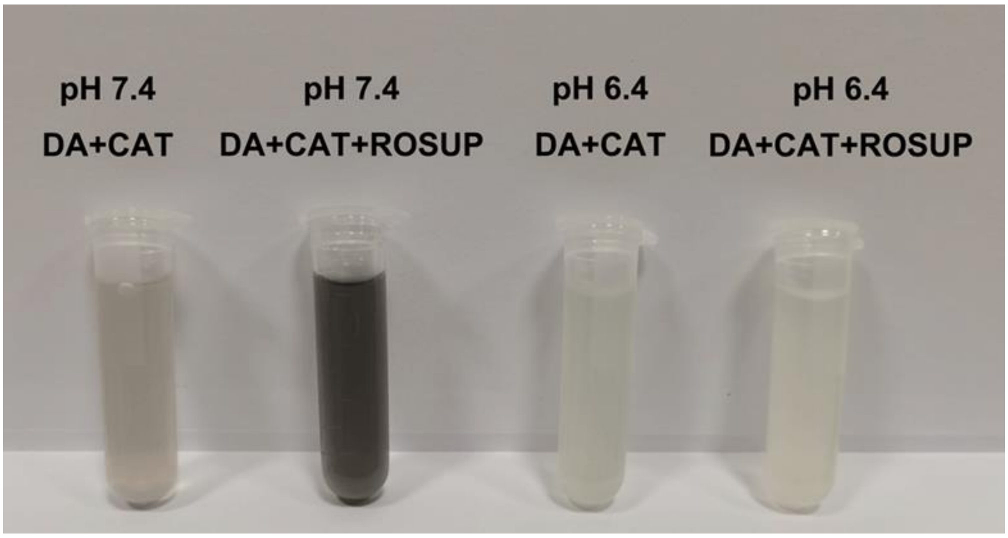

Figure S15. Incubation effects on CAT and DA Interaction. Images of incubation reactions between CAT (C_{CAT} = 1 mg/mL) and DA (C_{DA} = 1 mg/mL) in various citric acid-Na_2HPO_4 buffer conditions after 2 h.

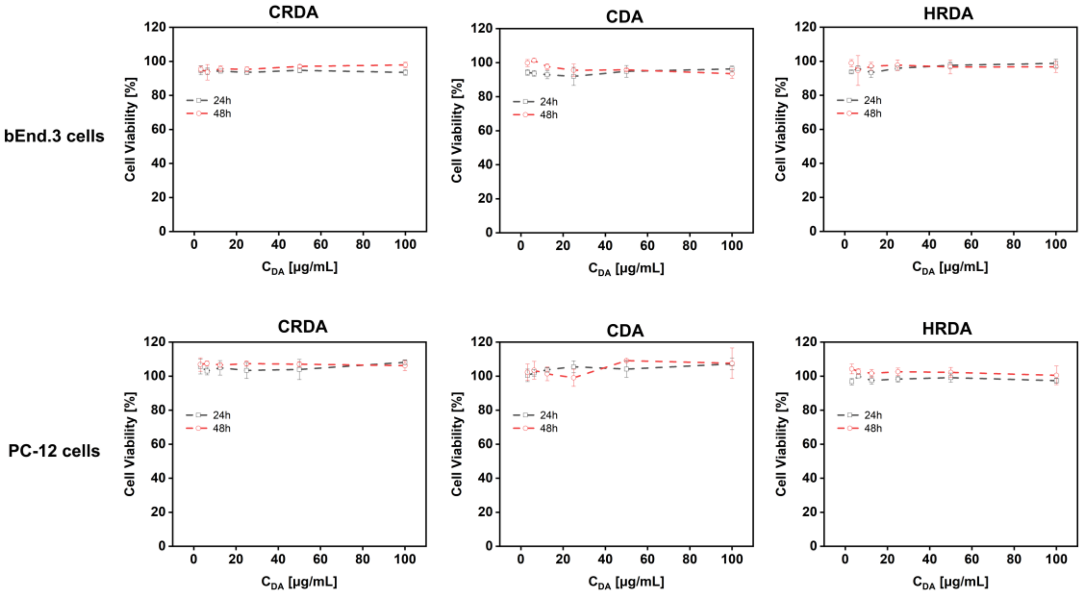

Figure S16. Cell Viability Studies. Cell viability (V) of bEnd.3 and PC-12 cells exposed to CRDA, CDA, and HRDA at different concentrations over 24 and 48 h, assessed using the resazurin assay. Data presented as mean ± standard deviation (n≥3).

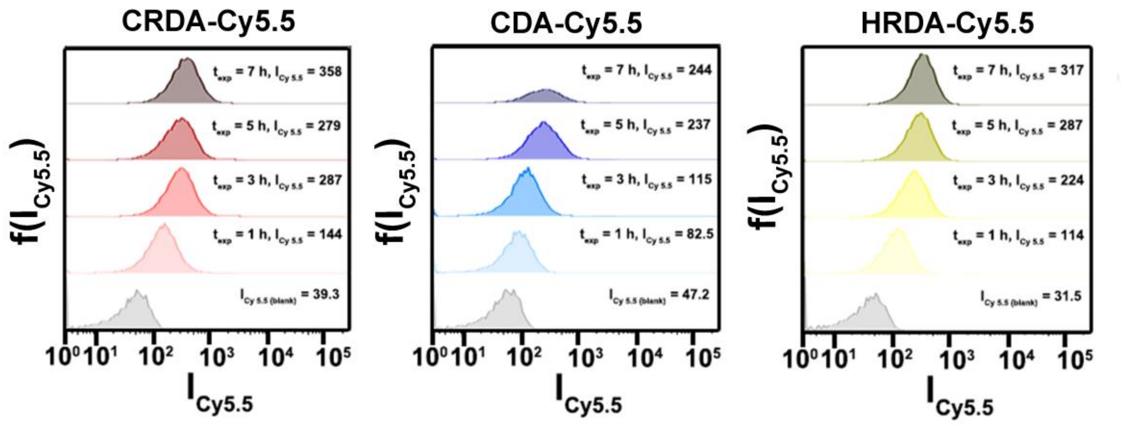

Figure S17. Cy5.5 Fluorescence Distribution. Distributions of Cy5.5 fluorescence per cell ($f(I_{Cy5.5})$) for bEnd.3 cells exposed to CRDA-Cy5.5, CDA-Cy5.5, and HRDA-Cy5.5 at C_{DA} = 50 μg/mL for 1, 3, 5, and 7 h, measured by flow cytometry.

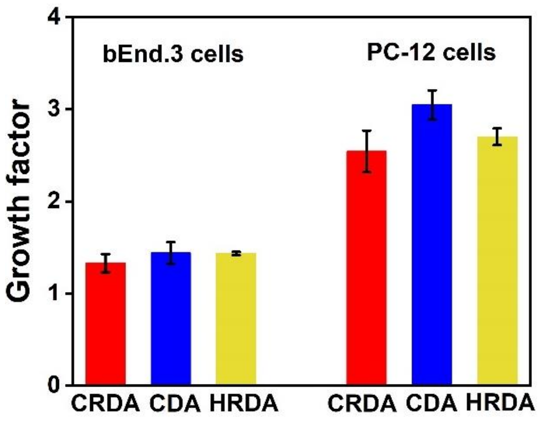

Figure S18. Compilation of data from Figure 2C. Proliferation factor of cells (N_{cell}) at t_{exp} = 5 h with t_{exp} + t_{inc} = 5 h + 24 h (n = 3). Absolute cell numbers at t_{exp} = 5 h provided as follow, 92.00 ± 8.63 ×10^4 cells/mL (CRDA), 90.25 ± 5.53 ×10^4 cells/mL (CDA) and 92.67 ± 5.03×10^4 cells/mL (HRDA).

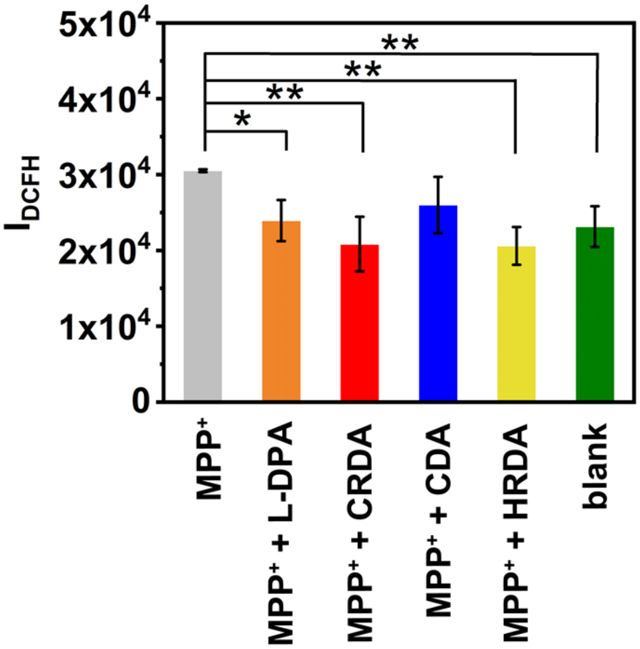

Figure S19. Compilation of data from Figure 3A. DCFH fluorescence (I_{DCFH}) in PC-12 cells exposed for t_{exp} + t_{inc} = 5 h + 24 h to L-DOPA (50 µg/mL), CRDA, CDA, and HRDA at C_{DA} = 50 µg/mL(n = 3). Significant differences analyzed using one-way ANOVA, **P < 0.05, **P < 0.01*.

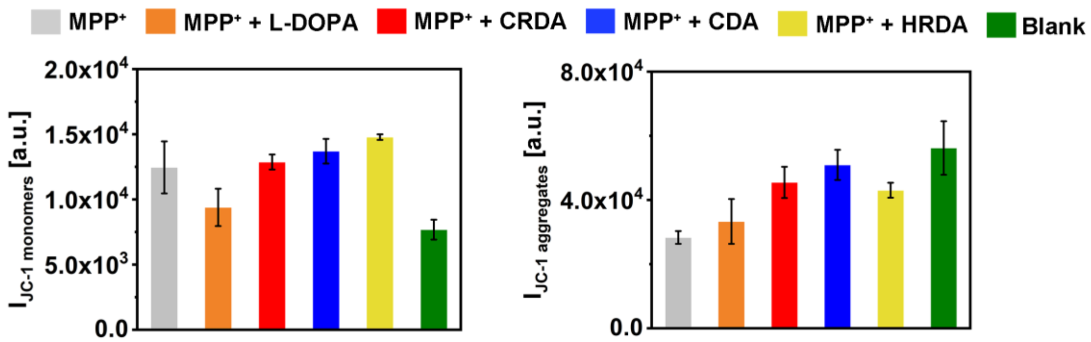

Figure S20. Compilation of data from Figure 3C. The fluorescence intensity of JC-1 monomers ($I_{JC\text{-}1\ monomers}$) and JC-1 aggregates ($I_{JC\text{-}1\ aggregates}$) in PC-12 cells following exposure to L-DOPA (50 µg/mL), CRDA, CDA, and HRDA at a concentration of C_{DA} = 50 µg/mL for a total of t_{exp} + t_{inc} = 5 h + 24 h. The results are from three independent samples (n = 3).

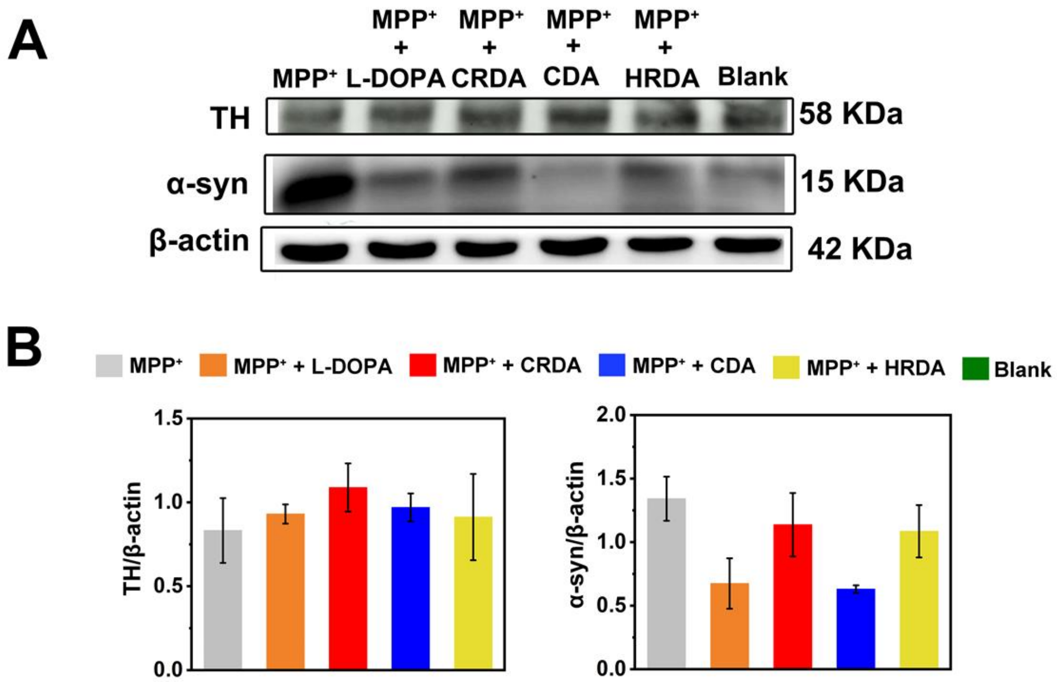

Figure S21. Compilation of data from Figure 3E. (A) Additional replicate of western blot displaying levels of tyrosine hydroxylase (TH) and alpha-synuclein (α-syn) in PC-12 cells treated under various conditions for $t_{exp} + t_{inc}$ = 5 h + 24 h. (B) Quantitative analysis of TH and α-syn expression across two replicates, conducted using Image J software. Expression levels were normalized to β-actin (n =2).

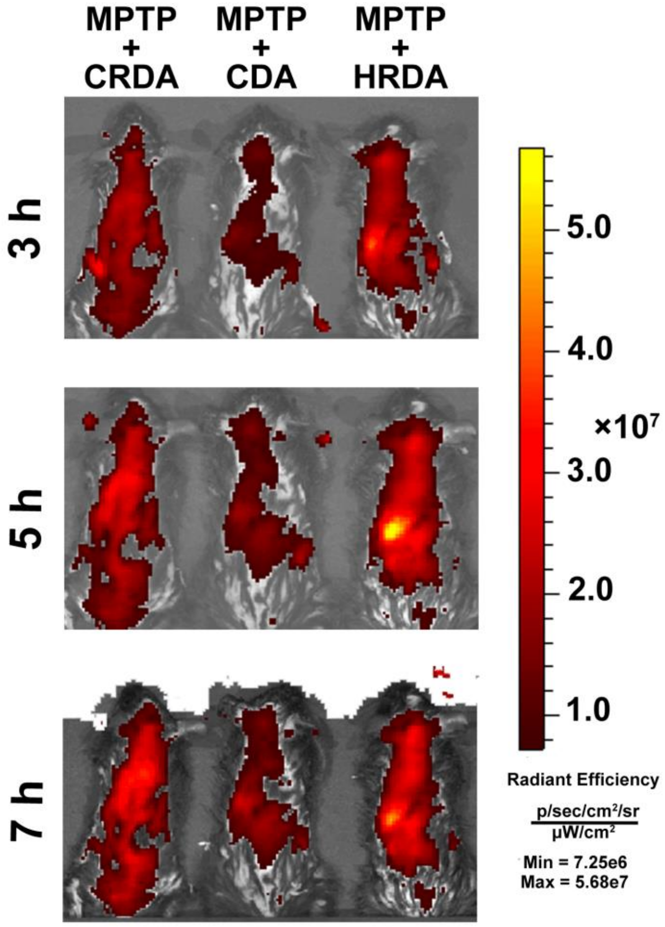

Figure S22. *In Vivo* Cy5.5 Fluorescence in MPTP Mice. Visualization of in vivo fluorescence in MPTP mice following intravenous injection of CRDA-Cy5.5, CDA-Cy5.5, and HRDA-Cy5.5 (C_{DA} = 600 µg/kg) at various time points (3, 5, and 7 h).

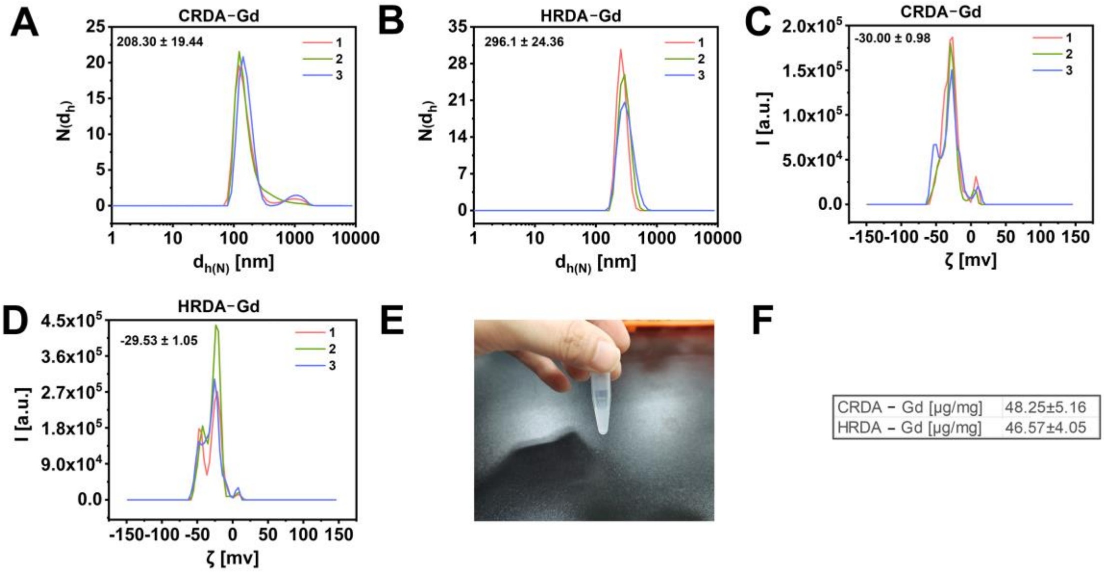

CRDA − Gd [μg/mg]	48.25±5.16
HRDA − Gd [μg/mg]	46.57±4.05

Figure S23. Characterizations of Gd Labeled NCs. (A-B) Hydrodynamic diameter distribution N(d_h) for CRDA-Gd (A) and HRDA-Gd (B) at a concentration of 1 mg/mL. (C-D) Zeta potential distribution I(ζ) for CRDA-Gd (C) and HRDA-Gd (D) at the same concentration. (E) Visual representation of CRDA-Gd in a milky white state. (F) Gadolinium (Gd) content within CRDA-Gd and HRDA-Gd quantified by ICP-MS.

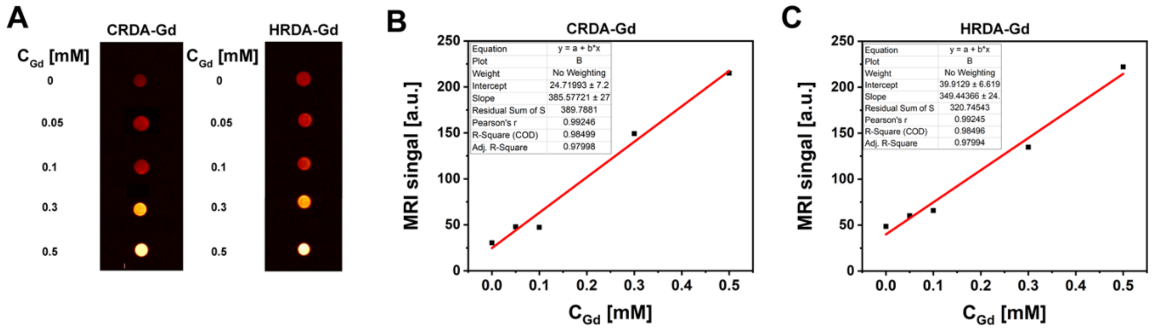

Figure S24. MRI Signaling of Gd-NCs. (A) MRI scanning of CRDA-Gd and HRDA-Gd at various concentrations. Linear fits for the MRI signal versus concentration for (B) CRDA-Gd (MRI signal = 385.58 × C_{Gd} + 24.72, R^2 = 0.98) and (C) HRDA-Gd (MRI signal = 349.44 × C_{Gd} + 39.91, R^2 = 0.98).

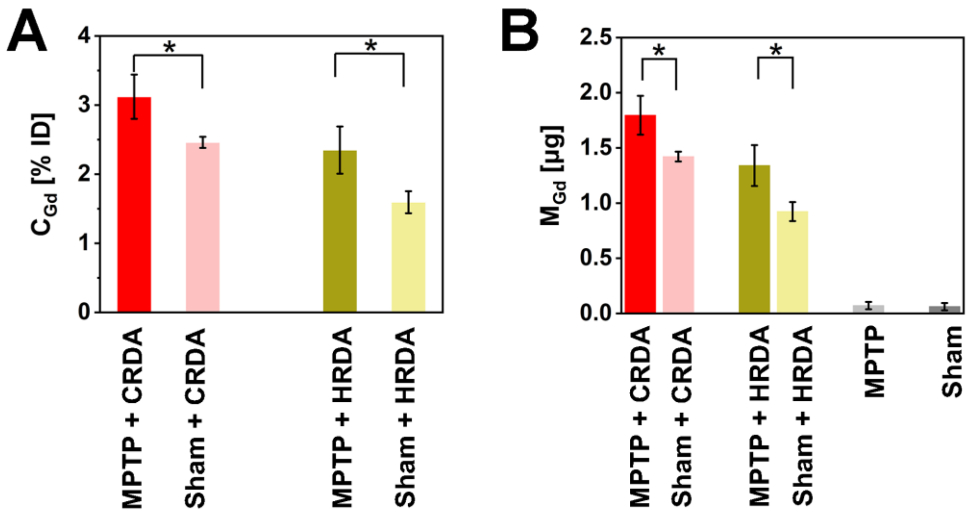

Figure S25. Alternate Data Representation from Main Manuscript Figure 4C. Displayed in units of (A) [% ID] and (B) [µg] for comparative analysis. The results are from three independent samples (n = 3). Significant differences analyzed using one-way ANOVA, *$*P < 0.05$.*

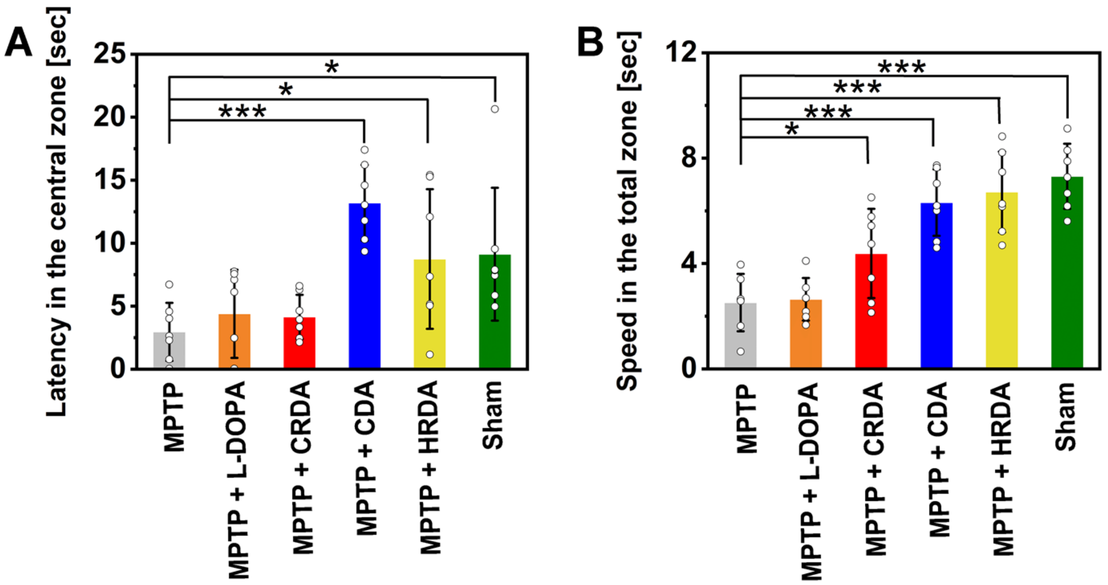

Figure S26. (A) Latency in the central zone and (B) speed in the total zone across of open field test all groups, with statistical significance determined by one-way ANOVA (n = 7, **P < 0.05, **P < 0.01, ***P < 0.001*).

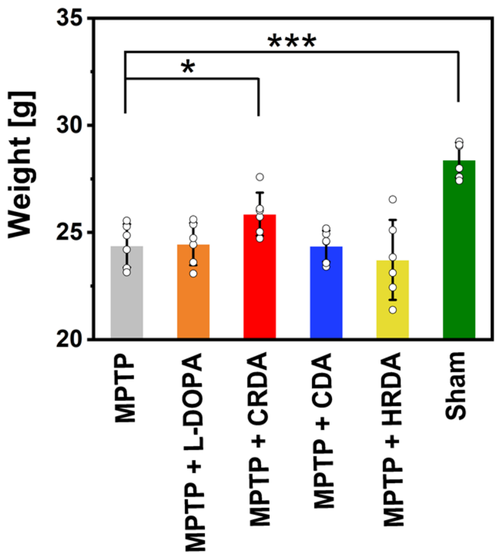

Figure S27. Group Weight Post-Behavior Test. Evaluation of the weight differences among all groups after the behavior test (n = 6), analyzed using one-way ANOVA for statistical significance, *$P < 0.05$, * *$P < 0.01$, * * * $P < 0.001$.

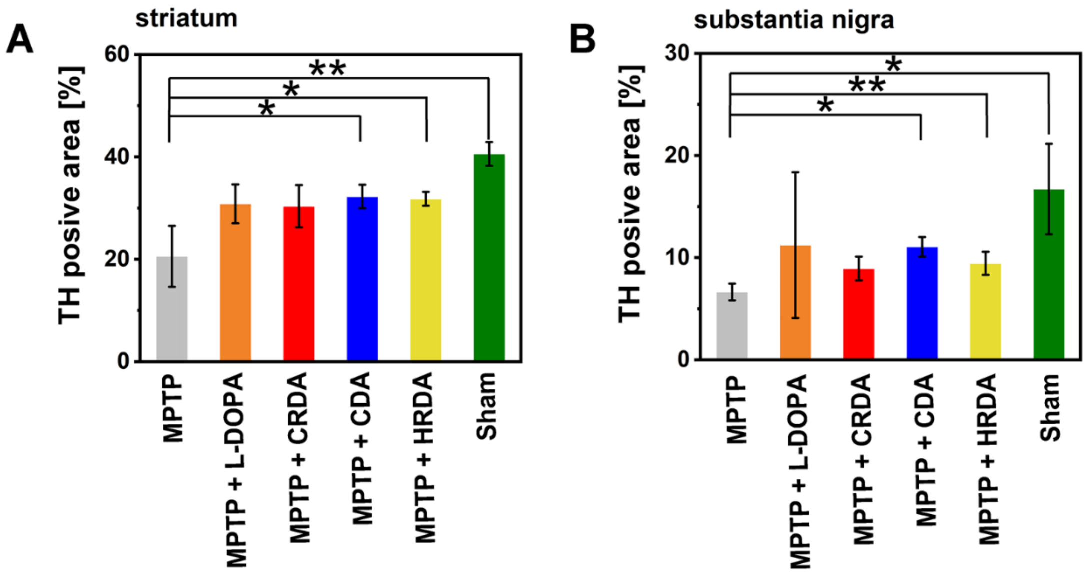

Figure S28. Compilation of data from Figure 7A showing TH positive areas in the (A) striatum and (B) substantia nigra, analyzed with Image J (n = 3). Significant differences analyzed using one-way ANOVA, *$^*P < 0.05$, $^{**}P < 0.01$.*

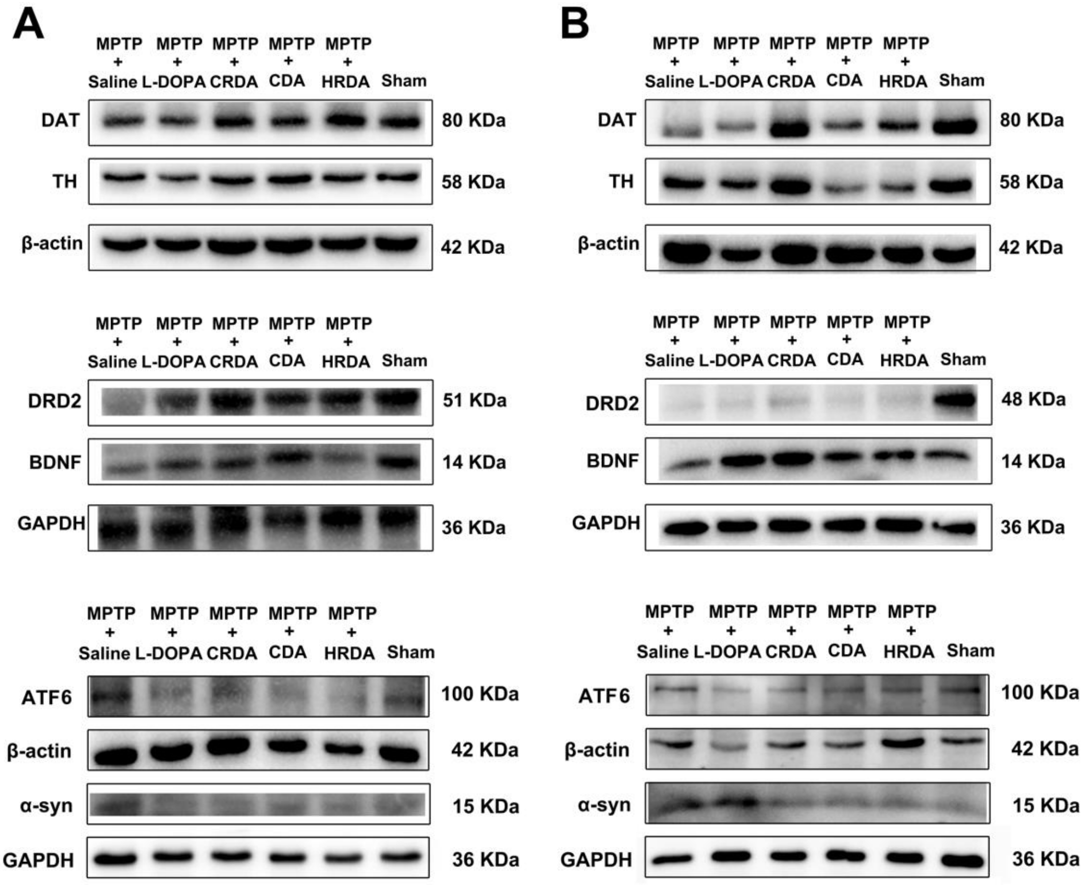

Figure S29. Compilation of data from Figures 7C, 7E, 8E, and 8G. The second replicate of western blot analysis for DAT, TH, DRD2, BDNF, ATF6, and α-syn in (A) the striatum and (B) the substantia nigra.

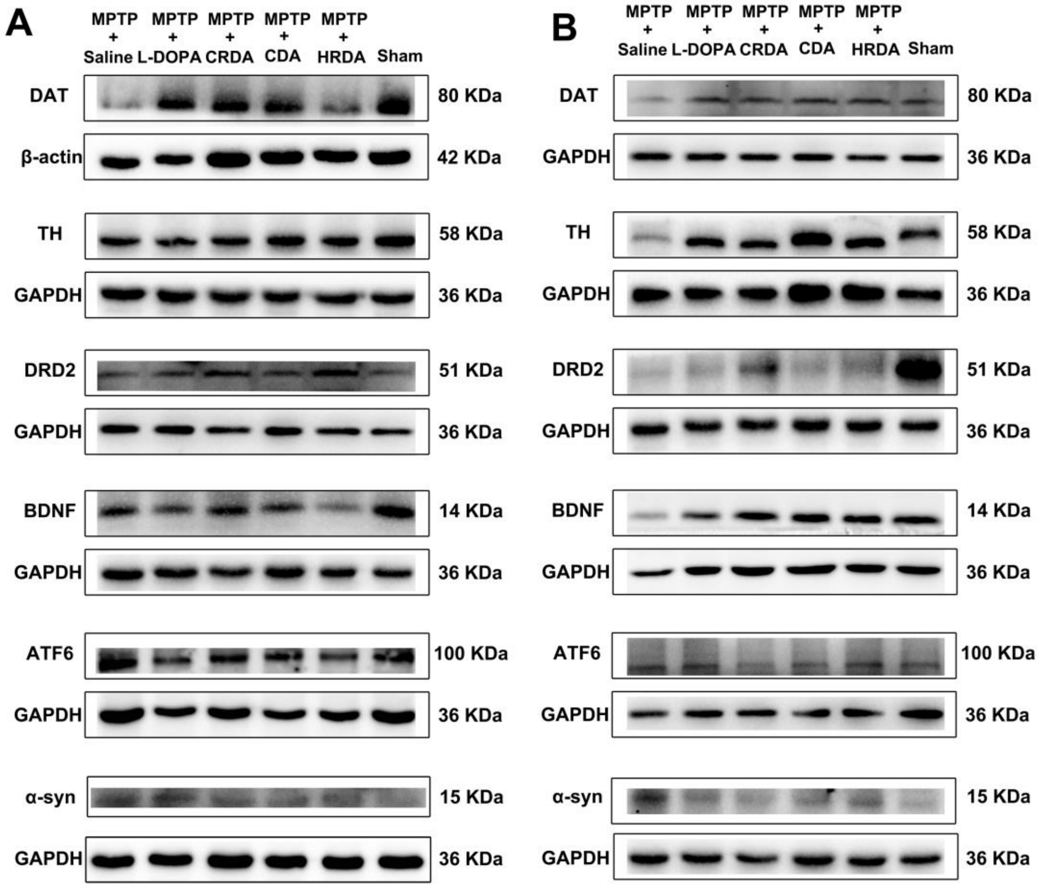

Figure S30. Compilation of data from Figures 7C, 7E, 8E, and 8G. The third replicate of western blot analysis for DAT, TH, DRD2, BDNF, ATF6, and α-syn in (A) the striatum and (B) the substantia nigra.

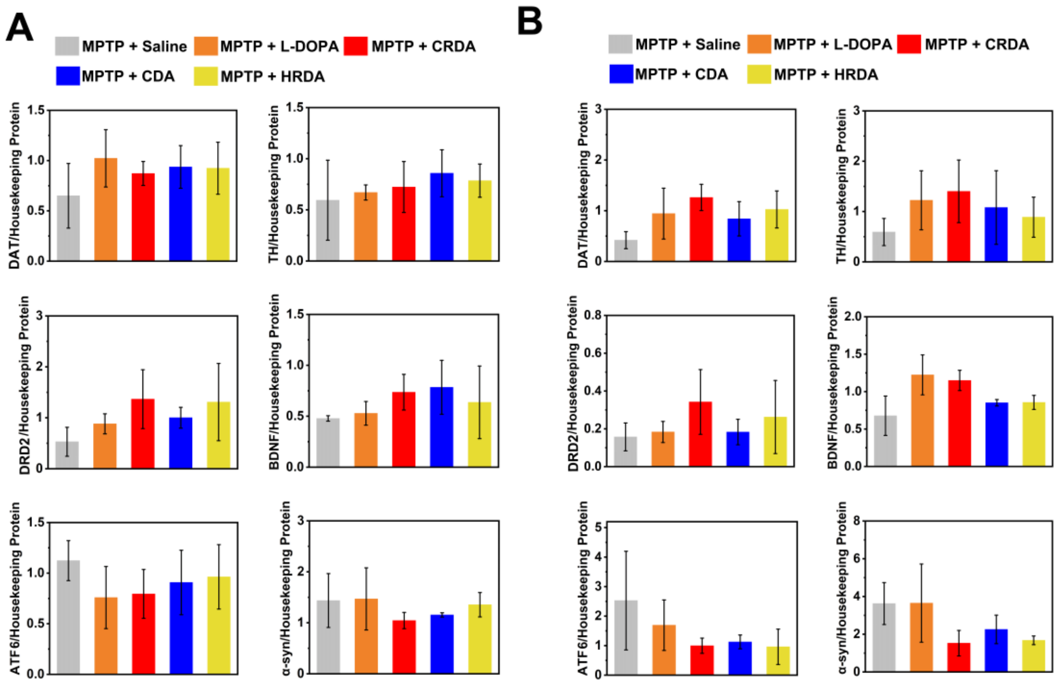

Figure S31. Quantitative analysis of grayscale detection for the western blot was performed using ImageJ. The ratio of target protein expression to housekeeping protein was calculated, and these ratios were further normalized to the sham group (n = 3).

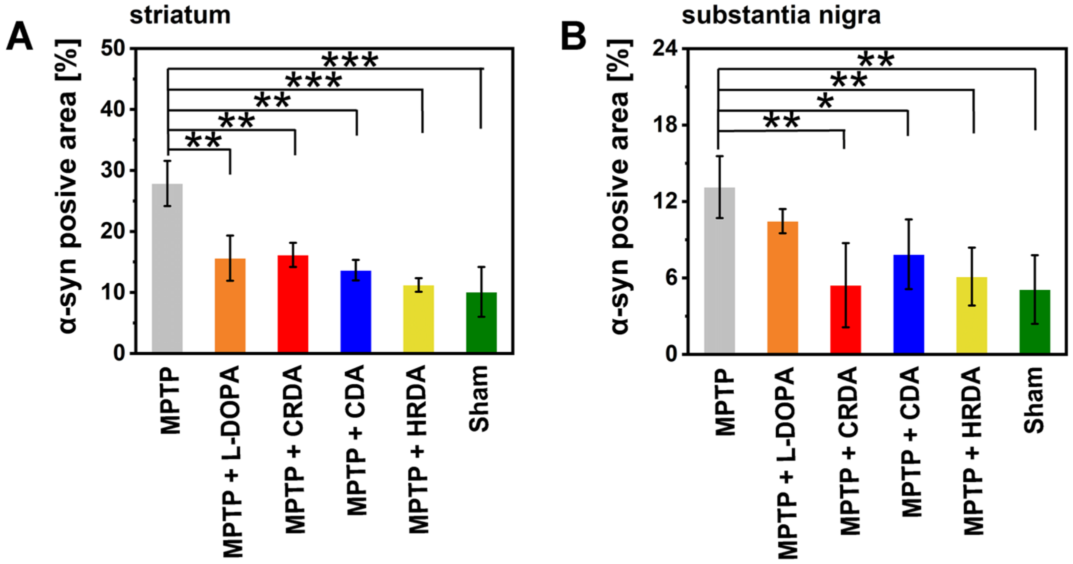

Figure S32. Compilation of data from Figure 8C showing α-syn positive areas in the (A) striatum and (B) substantia nigra analyzed with Image J (n = 3). Significant differences analyzed using one-way ANOVA, $^{*}P < 0.05$, $^{**}P < 0.01$. $^{***}P < 0.001$.

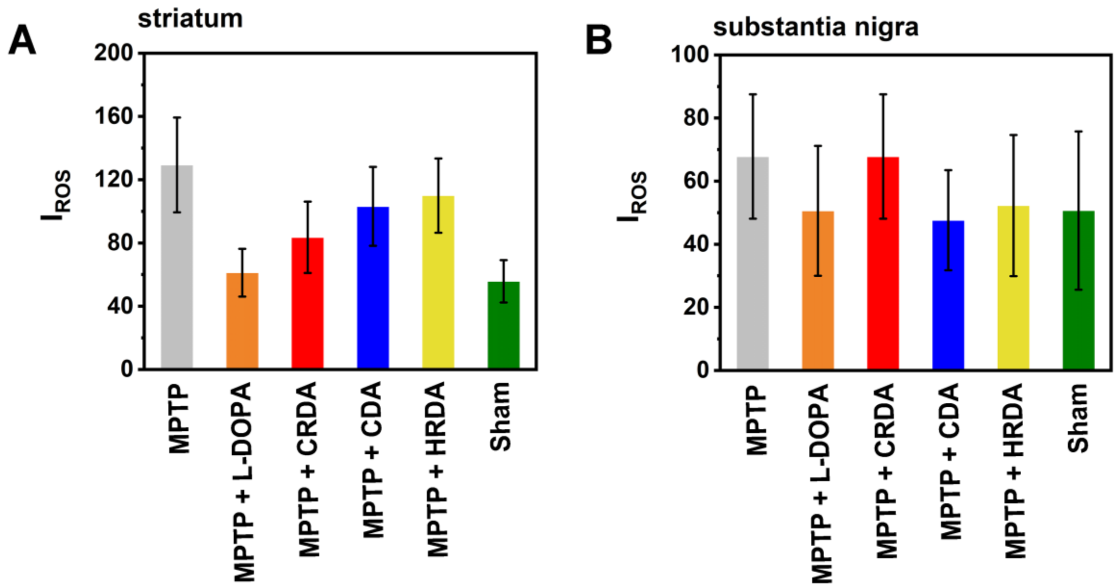

Figure S33. Compilation of data from Figure 8H showing ROS intensity in the (A) striatum and (B) substantia nigra, analyzed with Image J (n = 3).

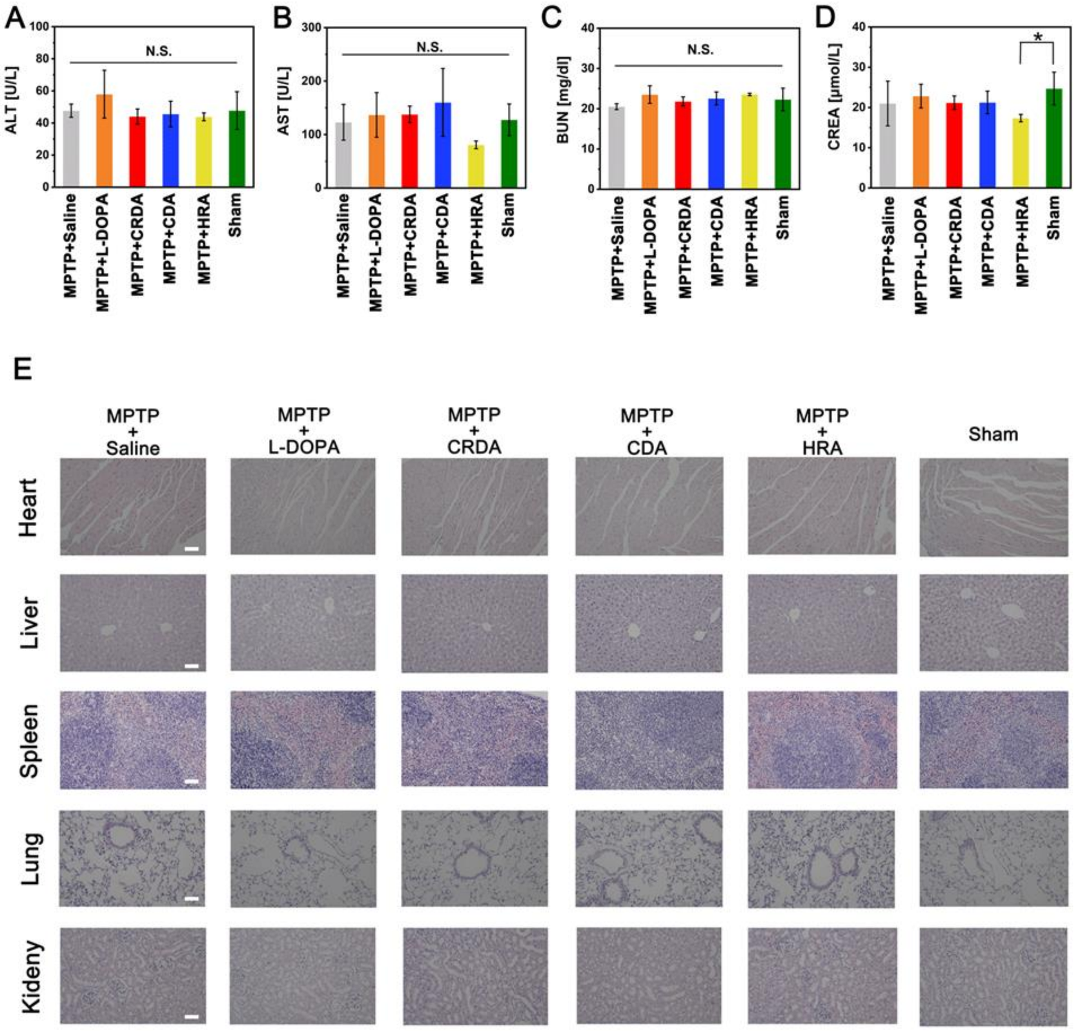

Figure S34. Evaluation of *In Vivo* Biocompatibility of NCs. The concentrations of liver and kidney biomarkers including (A) alanine aminotransferase (ALT), (B) aspartate aminotransferase (AST), (C) blood urea nitrogen (BUN), and (D) creatinine (CREA)in mice treated with various treatments (n = 3). Statistical analyses were conducted using one-way ANOVA, with "N.S." indicating no statistically significant differences among groups, $^{*}P < 0.05$. (E) Representative hematoxylin and eosin (HE) stained images of major tissues, including the heart, liver, spleen, lung, and kidney, in mice after various treatments. The scale bar represents 100 μm.
